# Limited Incremental Value of Six Haemogram-Derived Inflammatory Indices for Detecting Endometrial Carcinoma and Intraepithelial Neoplasia in Abnormal Uterine Bleeding: A Case–Control Study

**DOI:** 10.3390/diagnostics16193219

**Published:** 2026-10-05

**Authors:** Senem Karacabey Cakmak, Busra Seker Atas, Ezgi Gungor, Kubra Sevim Pehlivan

**Affiliations:** 1 Department of Obstetrics and Gynaecology, Haseki Training and Research Hospital, University of Health Sciences, 34265 Istanbul, Türkiye; bussraseker@hotmail.com; 2 Department of Obstetrics and Gynaecology, Denizli Çal State Hospital, 20700 Denizli, Türkiye; ezgi-gungor@outlook.com; 3 Department of Obstetrics and Gynaecology, Osmaniye Training and Research Hospital, University of Health Sciences, 80010 Osmaniye, Türkiye; sevimkubra247@gmail.com

**Keywords:** abnormal uterine bleeding, endometrial intraepithelial neoplasia, endometrial neoplasms, systemic inflammation response index, neutrophil-to-lymphocyte ratio, diagnostic accuracy, incremental value, case–control studies

## Abstract

**Background/Objectives:** Haemogram-derived inflammatory indices are proposed as inexpensive markers of endometrial neoplasia, but almost all supporting evidence is prognostic, from women already known to have carcinoma. We compared six indices head-to-head and asked whether any improved discrimination beyond a clinical model of age, menopausal status, endometrial thickness and metabolic comorbidity. **Methods:** In this single-centre retrospective case–control study, all women with endometrial intraepithelial neoplasia (EIN) or uterine malignancy on curettage for abnormal uterine bleeding (January 2021–June 2026) were identified (136 cases); 272 benign controls were drawn at a fixed 1:2 ratio. Indices were recalculated de novo from raw differential counts. Each log-transformed index was added singly to the fixed model; the likelihood-ratio test was the primary test and the change in C-statistic (ΔC) the effect measure, with ΔC ≥ 0.02 taken as clinically relevant, and performance was internally validated by bootstrapping and cross-validation. **Results:** Cases were 93 carcinomas and 43 EINs. Age (AUC 0.792, 95% CI 0.744–0.841) and endometrial thickness (0.749, 0.697–0.800) discriminated better than any index; SIRI was best (0.621, 0.564–0.678) and PLR did not discriminate (0.510). The clinical model reached a C-statistic of 0.882 (0.844–0.919), inflated by design; optimism-corrected calibration slope 0.963. No index remained significant after adjustment (smallest *p* = 0.141); the largest ΔC was +0.0042 (−0.0027 to +0.0111). The six log-indices are algebraically dependent, so the joint model used an identifiable four-index basis; it added +0.0064 (χ^2^_4_ = 4.48, *p* = 0.345), an increment that vanished on validation (optimism-corrected −0.0001; cross-validated +0.0010, −0.0076 to +0.0097). Accuracy at the Youden cut-off was 83.5% (clinical) and 81.7–84.2% (index models). Twelve sensitivity analyses agreed. **Conclusions:** None of the six indices provided detectable incremental discrimination once age, menopausal status and endometrial thickness were accounted for; their univariable associations reflect the clinical profile of affected women rather than an independent biological signal.

## 1. Introduction

Abnormal uterine bleeding (AUB) is among the most frequent reasons for gynaecological consultation, and corpus uteri cancer remains one of the most common gynaecological malignancies worldwide [1]. Under the FIGO PALM-COEIN system, AUB is attributed to structural and non-structural causes, with malignancy and hyperplasia among the structural group [2,3]. The clinical task the symptom poses is a triage problem: separating the majority of women with benign endometrium from the minority harbouring endometrial intraepithelial neoplasia (EIN) or carcinoma, entities now defined by the two-tiered WHO classification and addressed in current management guidance [4,5,6,7].

The prior probability of that minority diagnosis varies sharply with menopausal status. In postmenopausal bleeding, the pooled risk of endometrial cancer is 9% (95% CI 8–11), rising to 12% when hormone-therapy users are excluded [8], and reaching approximately 11% for EIN and carcinoma combined in postmenopausal bleeding in women aged 45 years or older [9]. In premenopausal AUB, the corresponding risk, including atypical hyperplasia, is only 1.31% (95% CI 0.96–1.80) [10]. Transvaginal endometrial thickness performs well as a rule-out test in symptomatic postmenopausal women, with a meta-analysis of 35 studies and 6302 patients reporting 95% sensitivity at a ≤4 mm threshold [11]. Endometrial sampling itself is imperfect: sensitivity is approximately 90% for carcinoma and 82% for atypical hyperplasia in postmenopausal bleeding, with sampling failure in 11% and insufficient material in 31% [12,13,14]. An inexpensive marker that could refine pre-procedural triage would therefore be genuinely useful.

Tumour-promoting inflammation is described by Hanahan and Weinberg as an enabling characteristic underpinning the hallmarks of cancer [15], an idea traceable to Virchow’s 1863 observation of leucocyte infiltration in neoplastic tissue [16]; the mechanisms now proposed in endometrial carcinogenesis include remodelling of the inflammatory microenvironment and immune escape [17]. The peripheral blood correlates—neutrophilia, lymphopenia, monocytosis and thrombocytosis—are captured in every complete blood count, and ratios derived from them have been studied extensively across solid tumours [18,19]. In endometrial cancer, NLR and PLR predict survival [20], SII is an independent prognostic factor [21,22], meta-analyses confirm the prognostic value of NLR, PLR and SIRI [23,24,25] and of SII [26], and PIV and SIRI have been examined in adjuvant and surgical cohorts [27,28,29,30]. RDW has attracted parallel interest [31,32,33,34].

Two features limit the clinical translation of this literature. First, the great majority of studies are prognostic: they enrol women in whom endometrial cancer has already been diagnosed and ask how long they will survive, rather than asking whether a woman presenting with bleeding harbours neoplasia. The smaller diagnostic literature is inconsistent—PLR discriminated endometrial pathology in one series while NLR did not [35], the reverse was reported in another [36], and reported AUCs range from 0.51 to 0.78 [37,38]; the inconsistency persists in recent series, in which inflammatory indices separated benign from malignant endometrium in one cohort but only the pan-immune-inflammation value survived multivariable adjustment [39], while none predicted concurrent carcinoma in endometrial intraepithelial neoplasia in another [40]; in the analogous problem of characterising adnexal masses a systematic review of ten studies found a pooled diagnostic AUC for NLR of only 0.66 [41]. Second, and more fundamentally, these studies compare indices with each other or with nothing at all. None asks the question that determines clinical usefulness: does the index add anything to the information the clinician already possesses when the decision to curette is made—age, menopausal status, transvaginal endometrial thickness and metabolic comorbidity? Recent models that report high discrimination do so by combining blood indices with clinical and tumour-marker variables, leaving the independent contribution of the inflammatory component unresolved [42,43,44,45].

This distinction matters because it determines what a positive result would license. An index that differs between pathology groups but conveys no information beyond age and endometrial thickness cannot change a triage decision, however small its *p*-value; conversely, a modest but genuinely independent contribution could be clinically worthwhile. Testing that requires a comparator model fixed on clinical grounds before the analysis, an outcome measure expressed as a change in discrimination rather than as an odds ratio, and a threshold for clinical relevance specified in advance. It also requires that the indices be examined together rather than singly, because they are collinear by construction: all six share the lymphocyte count in their denominator, and three of them additionally share the neutrophil count in their numerator, so any one of them can appear informative simply by proxying for the others.

We therefore compared six haemogram-derived indices head-to-head in women undergoing curettage for AUB and tested formally, as the primary question, whether any improved discrimination beyond a fixed clinical model containing the variables available at the moment the decision to curette is made. To our knowledge, this is the first study to frame the question as one of incremental value in a pre-curettage population.

## 2. Materials and Methods

### 2.1. Study Design and Setting

This was a single-centre, retrospective, unmatched case–control study conducted in the Department of Obstetrics and Gynaecology of Haseki Training and Research Hospital, University of Health Sciences, Istanbul, Türkiye, a tertiary referral centre. The study is reported in accordance with STROBE [46], TRIPOD [47,48] and STARD 2015 [49].

A protocol was written before data collection (v2.0) and revised after data collection but before the analyses reported here (v3.0). The revision was substantial, and eight of its changes were made after the primary results of the original analysis plan had been seen. These were: designation of incremental value as the primary endpoint; adoption of ΔC ≥ 0.02 as the threshold for clinical relevance; omission of the planned nomogram and decision curve analysis; projection of predictive values at external prevalences instead of reporting them from the sample; replacement of net reclassification indices by ΔC; abandonment of the original “enter variables with univariable *p* < 0.25” strategy in favour of a comparator model fixed on clinical grounds; replacement of a contradictory multiplicity scheme by Bonferroni for the primary tests with Benjamini–Hochberg for exploratory analyses; adoption of TRIPOD and STARD alongside STROBE; and elevation of the menopause-stratified analysis from a subgroup analysis to a central element of interpretation.

Sensitivity analyses SA9 to SA11 were added later still, after the asymmetries between the case and control groups had been identified during internal review. They were specified before being run, but not before the primary results were known.

This manuscript should therefore be read as a re-analysis under a revised plan, not as the execution of a plan fixed before the data were seen. Where the text describes a decision as fixed in advance, this means fixed in protocol v3.0 before the analyses reported here were run. The complete deviation log, with the timing of every change, is provided in Supplementary Table S1. Five further groups of analyses were added after the version first submitted, and therefore after its results were known: the calibration intercept and Brier score; bootstrap and cross-validated internal validation of both models; the identifiable specification of the joint index model and the corresponding correction of its degrees of freedom; the parallel chi-square tests of the categorical comparisons; and sensitivity analysis SA12. The first four were requested by reviewers. SA12 was added by the authors, on their own initiative, after an audit of the analysis dataset showed that three records failing the internal-consistency rule had been retained as entered (Section 2.4). None of the five was selected after inspecting its own result, and all are recorded with their timing in Supplementary Table S1.

### 2.2. Participants and Sampling

Eligible women were aged 18 years or older, underwent dilatation and curettage for AUB between January 2021 and June 2026, had an interpretable endometrial histopathology report, and had a complete blood count obtained within 30 days before the procedure. Exclusion criteria were haematological malignancy or myeloproliferative disease, active infection or antibiotic use within two weeks, systemic corticosteroid or immunosuppressive therapy, autoimmune disease, previous or concurrent malignancy of another organ, transfusion within one month, anticoagulant therapy, pregnancy-related bleeding, insufficient material, and cervical, endocervical or other non-endometrial malignancy. The period from January 2021 to June 2026 refers to the dates of the index curettage procedures recorded in the hospital archive, not to dates of enrolment; the study involved no patient contact and no study-specific procedure.

Cases were defined a priori as all women whose curettage specimen showed EIN or atypical hyperplasia, or endometrial carcinoma or another uterine malignancy; 162 such specimens were identified by complete enumeration across the whole period. Twenty-four showing cervical, endocervical or other non-endometrial malignancy were excluded, and two women initially classified as cases were subsequently found ineligible because no complete blood count had been obtained within 30 days before the procedure, leaving 136 cases.

Controls were sampled from the 1402 endometrial curettage specimens reported between January and June 2026 without a premalignant or malignant diagnosis. Two groups were identified and removed from the sampling frame before the draw: 47 specimens (3.4%) reported as insufficient for diagnosis, and 41 containing pregnancy-related material (products of conception or decidua). This left an eligible frame of 1314 specimens. From this frame, controls were drawn by simple random sampling with a recorded, reproducible seed (20260624) at the target ratio of two controls per case, giving 272 controls. No control was replaced after the draw. Patient flow is shown in Figure 1.

The non-histological exclusion criteria listed above were ascertained in both arms from coded diagnoses, admission records and discharge medication lists. Because these conditions are not systematically documented in routine records, no exclusion counts can be given for either arm and undocumented cases cannot be ruled out; this asymmetry between the histological criteria, which are documented, and the clinical criteria, which are not, is addressed in the limitations.

Because case and control ascertainment were separated in calendar time, this temporal mismatch is treated as the study’s principal design limitation and is quantified rather than argued around (Section 2.5).

Thirty-three cases (24.3% of all cases) were operated on during the SARS-CoV-2 pandemic period of 2021 and 2022, whereas no controls were operated on during this period. Under the institutional protocol in force at the time, every patient admitted for an elective procedure underwent preoperative SARS-CoV-2 PCR testing, and all patients included here tested negative. Active infection at the time of blood sampling is therefore excluded by documentation rather than by assumption. Residual effects of past infection or of vaccination on the differential count cannot be excluded by a negative PCR result, and this is addressed by a sensitivity analysis excluding all pandemic-period cases (SA9).

### 2.3. Variables

The outcome was the endometrial histopathological diagnosis, dichotomised into a benign group (normal, inactive or atrophic endometrium; proliferative endometrium; secretory endometrium; endometrial polyp; chronic endometritis; hyperplasia without atypia) and a premalignant or malignant group (EIN or atypical hyperplasia; endometrial carcinoma or other uterine malignancy). Hyperplasia was classified according to the two-tiered WHO 2020 system [4,5].

Candidate predictors were age, gravidity, parity, menopausal status, diabetes mellitus, hypertension, transvaginal endometrial thickness, and the complete blood count (haemoglobin, haematocrit, mean corpuscular volume, red cell distribution width, leucocytes, absolute neutrophil, lymphocyte, monocyte and eosinophil counts, platelets, mean platelet volume and platelet distribution width). Six indices constituted the pre-specified index set: NLR (neutrophils/lymphocytes), PLR (platelets/lymphocytes), MLR (monocytes/lymphocytes), SII (platelets × neutrophils/lymphocytes), SIRI (monocytes × neutrophils/lymphocytes) and PIV (neutrophils × platelets × monocytes/lymphocytes). Red cell distribution width and mean platelet volume were examined separately and exploratorily as non-ratio haemogram parameters that have attracted independent interest in this literature [31,32,33,34]; they are not part of the primary hypothesis and are reported as such. Body mass index was recorded but was missing in 217 of 408 women (53.2%); in accordance with the protocol’s pre-specified rule that variables missing in more than 20% be excluded, it was not entered into the main models and was examined only in a sub-cohort analysis. Bleeding pattern, menopause duration, polycystic ovary syndrome, smoking, tamoxifen, hormone replacement therapy and family cancer history could not be retrieved reliably from retrospective records and are treated as unmeasured confounders.

Exclusion criteria other than histological ones were ascertained from admission records, discharge medication lists and coded diagnoses. Because these conditions are not systematically documented, undocumented cases cannot be ruled out. Specimens reported as insufficient were removed from the sampling frame before the draw (*n* = 47), and no such specimen therefore entered the analysed cohort; this is addressed in the limitations.

### 2.4. Data Quality Control

The raw dataset was frozen and never overwritten; all analyses were performed on an analysis dataset derived from it by a written rule set (Supplementary Table S2). All indices were recalculated de novo from raw differential counts rather than accepted as entered. Each record was required to satisfy range checks and an internal consistency rule requiring the summed absolute differential (neutrophils + lymphocytes + monocytes + eosinophils) to fall between 0.85 and 1.15 times the total leucocyte count. Failing this rule flagged a record for individual inspection; it was not a rule for automatic exclusion, and the disposition of every flagged record is given in the repair log (Supplementary Table S2).

Sixteen records violated the consistency rule and were inspected individually. In eight, differential values had been entered as percentages rather than absolute counts; these were converted using the recorded leucocyte count, and the consistency ratio after repair ranged from 0.993 to 0.999. Two records showed a decimal shift in the monocyte count. One leucocyte count was typographically wrong and was corrected; it does not enter any index. In two records the responsible cell could not be identified unambiguously and the indices derived from them were themselves biologically implausible—a lymphocyte count of 0.05 × 10^3^/µL giving an NLR of 62.8 and a PLR of 5520, and a monocyte count equal to the lymphocyte count giving an MLR of 1.00 against a cohort range of 0.08 to 0.79—and all six indices were set to missing in these two rather than repaired. The remaining three flagged records, with consistency ratios of 1.16, 1.28 and 1.44, were retained as entered. In each of the three, the summed differential exceeded rather than fell short of the leucocyte count, and the leucocyte count entered none of the six indices; every index derived from these records lay inside the cohort distribution (NLR 1.66 to 2.61, PLR 76 to 122, MLR 0.107 to 0.247, SIRI 0.81 to 1.21). In one of the three, the recorded leucocyte count was identical to the recorded neutrophil count, so the possibility that a field other than the leucocyte count was in error cannot be excluded; SA12 is the test of exactly that contingency. Retaining them is a judgement rather than a rule, and it is tested rather than asserted: setting their indices to missing as well is reported as a sensitivity analysis (SA12). Four implausible values in variables outside the index set (MPV 0.30 fL, RDW 3.6%, MCV 225 fL, haematocrit 12.8%) were set to missing. Two RDW values of 27.0% and 27.1% were retained because they were accompanied by microcytosis and anaemia consistent with genuine iron deficiency, and four endometrial thickness values of 42–45 mm were retained because all occurred in postmenopausal women aged 65–75 with carcinoma. After repair, all six indices were computable in 405 of 408 women (99.3%). Missing values arising in the source records are reported separately from values set to missing during cleaning in Supplementary Table S8; the two sources are distinguished because only the latter appear in the repair log. Five haemoglobin values and one lymphocyte count were absent from the source records; all other missingness in the haemogram was introduced by the cleaning rules.

### 2.5. Statistical Analysis

All continuous variables departed from normality and are summarised as median (interquartile range) and compared with the Mann–Whitney U test, with the rank-biserial correlation and its bootstrap 95% confidence interval as the effect size. The magnitude of the rank-biserial correlation is described throughout using the conventional correlation benchmarks applied by analogy—|r| < 0.10 negligible, 0.10 to <0.30 small, 0.30 to <0.50 moderate and ≥0.50 large—and these descriptors are used consistently in Section 3. These bands are conventions rather than derivations, and they are not the only ones proposed: Funder and Ozer [50] argue for a less stringent scale on which a correlation of 0.20 is already medium, and under that scale every index would move one band higher. The relative ordering that carries the argument here would be unaffected, since age and endometrial thickness exceed every index by a factor of two or more on either scheme; the descriptors are therefore used as a common vocabulary and not as evidence. Categorical variables are given as n (%). Every 2 × 2 comparison was tested both by Fisher’s exact test and by Pearson’s chi-square test without continuity correction, and the minimum expected cell frequency is reported for each (Supplementary Table S10). Fisher’s exact test is the exact conditional test and is valid at any expected frequency, whereas the chi-square statistic is its large-sample approximation and requires adequate expected counts; the two are therefore not alternatives selected by cell size, and the exact test is not restricted to sparse tables. The conventional expected-count rule has itself been questioned from both directions: Campbell [51] shows that the N−1 chi-square remains accurate down to a minimum expected count of 1, whereas Lydersen and colleagues [52] hold that Fisher’s exact test is conservative and recommend unconditional exact or mid-p tests in preference to it. In this cohort, the minimum expected frequency was 56.0, 20.7, 28.3 and 7.0 for postmenopausal status, diabetes, hypertension and nulliparity, but 1.67 for hysteroscopy-guided procedures, where the chi-square approximation is not appropriate. Both tests were computed and agreed on every variable, so the choice affects no conclusion; Fisher’s exact *p*-values are the ones reported for the categorical variables because they remain valid for every row, including the sparse one, and a single test should be reported for all categorical variables.

Discrimination for each continuous predictor was quantified by the AUC with a DeLong confidence interval, and an optimal threshold was identified by the Youden index, with sensitivity and specificity reported with Wilson intervals and likelihood ratios. Pairwise comparisons between indices used DeLong tests.

The multivariable analysis was structured to answer the incremental-value question. The clinical model (Model A) contained the variables available before curettage—age, menopausal status, endometrial thickness, diabetes and hypertension. This model was fixed on clinical grounds and deliberately not selected by significance testing; as noted in Section 2.1, that decision replaced an earlier significance-based strategy and was taken after the primary results of the original plan had been seen. No variable was therefore admitted to or excluded from Model A on the basis of a univariable *p*-value. Nulliparity, RDW and MPV as univariable descriptors only and were never candidates for Model A. Conversely, all six indices, including PLR, were carried into the primary analysis irrespective of univariable significance, because the pre-specified question was whether any member of a fixed index set adds incremental value; dropping the non-significant members would have converted a pre-specified head-to-head comparison into a data-driven selection, would have invalidated the Bonferroni correction for six tests, and would have removed the one index whose null result is itself informative. Because all six indices share lymphocytes in their denominator, and SII, SIRI and PIV additionally share neutrophils in their numerator, they are collinear by construction and were never entered together except in the joint model reported below [53]. That collinearity is not merely strong but exact. All six indices are multiplicative functions of only four cell counts, so their logarithms satisfy two identities: ln(SII) = ln(PLR) + ln(SIRI) − ln(MLR) and ln(PIV) = ln(SII) + ln(SIRI) − ln(NLR). The six log-indices consequently span a four-dimensional space; a design matrix containing all six is rank-deficient, and the individual coefficients of all six are not identifiable [54]. The joint model (Model H) was therefore specified with a maximal identifiable basis of four log-indices—NLR, PLR, MLR and SIRI—which reproduces the fitted values, the C-statistic and the likelihood-ratio statistic of the six-term specification exactly while restoring identifiability of the individual terms, and whose likelihood-ratio test carries four rather than six degrees of freedom. Each index was log-transformed and added singly to Model A (Models B–G).

The inferential procedure follows Demler and colleagues, who showed that for strictly nested models the null distribution of the difference in areas under the curve is degenerate rather than normal, so that the DeLong test is invalid in this setting and behaves conservatively, with type I error far below nominal and correspondingly low power [55]. Their recommendation, which is also that of Vickers and colleagues [56], is to test the added predictor by a single conventional test of association and, only if that test is significant, to estimate the change in discrimination with a confidence interval. We therefore report the likelihood-ratio test of the index term as the primary inferential test, with the Wald test alongside it. The change in C-statistic (ΔC) is retained as the descriptive effect measure and is reported with a DeLong confidence interval, which Demler and colleagues endorse for this purpose; the DeLong *p*-value is also given, but only for comparability with the prior literature, and it should be read as conservative. One caveat should be stated: Demler and colleagues validate DeLong-based intervals in the non-degenerate case and do not evaluate their coverage under the null, which is the situation encountered here, so the intervals reported for ΔC should be treated as approximate. Because no index term reached significance, these intervals are not offered as estimates of a real incremental gain; they are reported to define the upper bound the data can exclude, which is the quantity this study was designed to establish.

A ΔC of at least 0.02 was adopted as the threshold for clinical relevance: the smallest change we judged capable of altering a triage decision. The choice is a clinical judgement rather than a statistical derivation and was fixed in protocol v3.0 before the analyses reported here were run; three considerations support it. First, the C-statistic is an insensitive measure of the value added by a new marker: a predictor may be strongly associated with the outcome and still leave discrimination almost unchanged [57], so a lower threshold would classify as relevant differences that could not plausibly move a triage decision. Second, published models for endometrial cancer risk span C-statistics of 0.56 to 0.92 [58], so a candidate marker must displace a comparator that is already strong. Third, the threshold is a stringent bar against a comparator with a C-statistic of 0.882, since ΔC is bounded above by 0.118 and increments of this size are rarely achieved when a biomarker is added to a strong clinical model; 0.02 therefore represents 17% of the entire remaining discriminatory space, which is a demanding rather than a lenient bar. All estimates are reported with confidence intervals so that readers applying a different threshold can judge for themselves, and the upper confidence limits are stated explicitly in the Results so that the threshold can be varied without re-analysis. We note that a change in the C-statistic is not itself a measure of clinical utility; net benefit would be preferable, but it cannot be estimated from case–control data alone, because the prevalence is fixed by the sampling ratio and must be supplied from an external source before net benefit can be computed [59], as set out below.

Diagnostic measures were computed for every model so that discrimination could also be read on the scale on which a triage decision is made. Predicted probabilities were obtained from the fitted model, a cut-off was identified by the Youden index exactly as for the individual predictors above, and sensitivity, specificity and overall accuracy were computed at that cut-off with Wilson confidence intervals. Two qualifications are built into the reporting. A cut-off that maximises the Youden index in the sample in which it is estimated is optimistic, so accuracy is additionally reported after bootstrap optimism correction, obtained by re-estimating both the model and its cut-off in each of 500 replicates and applying them to the original data. And because the resulting cut-offs differ between models, the corresponding sensitivity–specificity pairs do not lie at a common operating point and cannot be compared directly; every model was therefore also evaluated at the single cut-off of the clinical model, at which the two classifications are paired within the same women and can be compared by McNemar’s test in its exact binomial form [60]. Accuracy is a function of prevalence, and the sample prevalence is fixed at 33.3% by the sampling ratio, so these quantities describe classification within this cohort and are not estimates of performance in a population with a different prevalence.

Because the study analyses a fixed, complete enumeration of cases, no a priori power calculation was performed; the relevant design quantity is precision [61]. The number of cases was not chosen: it is every eligible premalignant or malignant specimen in the institutional archive for the study period, so a target sample size could not be set and a power calculation would have been post hoc. The control-to-case ratio was fixed a priori at 1:2 because the variance of a case–control comparison falls steeply between 1:1 and 1:2 and only marginally thereafter, so further controls would have added cost without material precision [62]. With 136 cases and 272 controls, the expected half-width of the 95% confidence interval is 0.059 for an AUC of 0.60, 0.053 at 0.75 and 0.040 at 0.88, and the observed half-width for ΔC was 0.007. Adequacy for model development was checked against the criteria of Riley and colleagues: with five candidate parameters and the observed Cox–Snell R^2^ of 0.381, a minimum of 91 participants is required to keep expected shrinkage below 10%, whereas 408 participants with 136 events were available, giving 27.2 events per candidate parameter [61]. The design can therefore exclude an incremental gain above approximately 0.015 but cannot resolve smaller ones.

Model performance was assessed for both discrimination and calibration. Optimism-corrected C-statistics and calibration slopes were obtained by bootstrap resampling with 500 replications [53,63]. Calibration was quantified at two levels of the calibration hierarchy: mean calibration, or calibration-in-the-large, estimated as the calibration intercept by refitting an intercept-only model with the linear predictor as a fixed offset, and weak calibration, estimated as the calibration slope [64,65]. Each bootstrap replicate was used to refit the model, which was then applied to both the bootstrap sample and the original data; the mean difference was subtracted from the apparent estimate of the C-statistic, the calibration slope, the calibration intercept and the Brier score [66]. Because bootstrap optimism correction and cross-validation answer slightly different questions, 10-fold cross-validation repeated 20 times was performed in addition, and performance was recomputed on the pooled out-of-fold linear predictors [67]. The complete regression equation of the clinical model, including its intercept, is reported so that it can be applied or checked externally. Nagelkerke R^2^ and the Hosmer–Lemeshow test are reported [68]. Complete-case analysis was used for variables missing in under 5%; body mass index, missing in 53.2%, was excluded from the main models under the protocol’s pre-specified rule, and because its missingness differed between groups, the mechanism cannot be assumed to be at random and imputation was not attempted [69].

Because cases and controls were sampled at a fixed 1:2 ratio, the sample prevalence does not reflect the source population; absolute risk estimates, calibration-in-the-large and net benefit are therefore not identifiable, and no nomogram or decision curve analysis was undertaken [59,62,70]. Predictive values are instead projected at realistic prevalences of 2%, 5% and 11% and reported in Supplementary Table S3.

Effect modification by menopausal status was tested by adding a product term to each index model, and stratum-specific analyses were treated as central to interpretation rather than as subgroup analyses, because cases and controls were not matched on age. Eleven sensitivity analyses were specified before being run (SA1–SA11), and a twelfth (SA12) was added during revision: restriction of cases to carcinoma alone (SA1); exclusion of hysteroscopy-guided procedures (SA2); exclusion of every record touched by data repair (SA3); adjustment for the blood count-to-curettage interval (SA4); a temporal drift probe within the case group together with restriction of cases to 2025–2026 (SA5); addition of body mass index in the sub-cohort in which it was recorded (SA6); trimming of the extreme 1% of each index (SA7); penalised regression (SA8); exclusion of pandemic-period cases (SA9); restriction to the age range common to cases and controls, 45 to 65 years (SA10); comparison of women with and without a recorded body mass index (SA11); and, added during revision after an audit of the analysis dataset, setting to missing the indices of the three records that failed the internal-consistency rule but were retained as entered (SA12). The prefix SA distinguishes these analyses from the supplementary tables, which are numbered S1 to S10 (with S7b).

For the six primary index tests, a Bonferroni threshold of *p* < 0.0083 was applied; exploratory and sensitivity analyses used Benjamini–Hochberg correction, with uncorrected *p*-values also reported. Two-sided *p* < 0.05 was the nominal significance level. Analyses used Python 3.11 with pandas, SciPy, statsmodels, scikit-learn and matplotlib; DeLong variance estimation [71] followed the fast algorithm of Sun and Xu [72]. A generative artificial intelligence tool was used in an advisory capacity during literature searching and during review of the statistical analysis plan; it did not generate data, perform the analyses or select the results reported. All analyses were executed and verified by the authors, and every reference was checked individually at DOI or PMID level. Product details are given in the Acknowledgements.

### 2.6. Ethics

The study was approved by the institutional Clinical Research Ethics Committee (decision no. 131-2026, dated 13 May 2026), which authorised retrospective review of the records of women who underwent endometrial curettage between 1 January 2021 and 30 June 2026 and granted a waiver of individual informed consent owing to the retrospective design and the exclusive use of anonymised routine clinical data. Hospital records were accessed, and the analysis dataset was extracted only after this approval, beginning on 18 May 2026; the control sampling draw was executed on 24 June 2026. No patient was contacted, and no data were generated for the purposes of the study. All data were anonymised before analysis, and the study was conducted in accordance with the Declaration of Helsinki.

## 3. Results

### 3.1. Cohort and Histopathology

All 408 sampled women were analysed: 136 cases and 272 controls (Figure 1). Baseline characteristics are given in Table 1 and the distribution of histopathological diagnoses in Table 2.

**Table 1:** Baseline characteristics of the analysed cohort. Continuous variables are median (interquartile range); categorical variables are n (%). Effect size is the rank-biserial correlation with a bootstrap 95% confidence interval for continuous variables, and the odds ratio for categorical variables. *p*-values are from the Mann–Whitney U test for continuous variables and Fisher’s exact test for categorical variables. Both Fisher’s exact test and Pearson’s chi-square test were computed for every categorical variable; the two agreed on all five, and the chi-square statistics with their minimum expected cell frequencies are given in Supplementary Table S10. Effect sizes for continuous variables carry the magnitude descriptor defined in Section 2.5. Odds ratios use the Haldane-corrected Woolf method and therefore differ marginally from the logistic-regression estimates reported in the multivariable analyses. Where a variable was not available in all 408 women, the number analysed is given in parentheses; Supplementary Table S8 separates missingness present in the source records from values set to missing during data cleaning.

Variable	All (n = 408)	Benign (n = 272)	Premalignant/ Malignant (n = 136)	*p*	Effect Size (95% CI)
Age, years	49.00 (43.00–59.00)	46.00 (41.00–51.00)	60.00 (50.00–67.00)	<0.001	0.58 (0.49 to 0.68), large
Body mass index, kg/m^2^ (n = 191)	29.40 (25.10–34.05)	27.10 (24.10–31.45)	33.20 (28.30–37.17)	<0.001	0.43 (0.28 to 0.56), moderate
Gravidity	3.00 (2.00–5.00)	3.00 (2.00–4.00)	3.00 (2.00–5.00)	0.326	0.06 (−0.06 to 0.18), negligible
Parity	2.00 (2.00–4.00)	2.00 (2.00–3.00)	3.00 (2.00–4.00)	0.212	0.07 (−0.04 to 0.19), negligible
Endometrial thickness, mm	10.00 (7.00–14.00)	8.00 (6.00–12.00)	14.00 (10.00–20.00)	<0.001	0.50 (0.40 to 0.60), large
CBC-to-curettage interval, days	3.00 (1.00–15.00)	2.00 (1.00–11.25)	7.50 (1.00–20.00)	0.005	0.17 (0.04 to 0.29), small
Haemoglobin, g/dL (n = 403)	12.80 (11.60–13.60)	12.80 (11.65–13.60)	12.75 (11.47–13.90)	0.851	0.01 (−0.11 to 0.13), negligible
MCV, fL (n = 407)	86.10 (81.70–89.30)	86.05 (81.45–89.10)	86.30 (82.05–89.30)	0.634	0.03 (−0.09 to 0.15), negligible
RDW, % (n = 407)	13.70 (13.10–15.10)	13.70 (13.00–15.10)	13.80 (13.20–15.03)	0.334	0.06 (−0.05 to 0.17), negligible
Leucocytes, ×10^3^/µL	7.42 (6.16–8.62)	7.17 (6.05–8.36)	7.79 (6.52–9.44)	0.005	0.17 (0.05 to 0.29), small
Neutrophils, ×10^3^/µL (n = 406)	4.38 (3.59–5.57)	4.19 (3.44–5.33)	4.79 (3.80–6.10)	<0.001	0.22 (0.10 to 0.32), small
Lymphocytes, ×10^3^/µL (n = 405)	2.16 (1.74–2.70)	2.16 (1.76–2.70)	2.17 (1.71–2.71)	0.600	−0.03 (−0.15 to 0.09), negligible
Monocytes, ×10^3^/µL (n = 406)	0.43 (0.35–0.53)	0.42 (0.34–0.50)	0.45 (0.38–0.56)	0.009	0.16 (0.04 to 0.27), small
Platelets, ×10^3^/µL	271.50 (229.00–316.00)	271.00 (231.75–313.50)	275.00 (222.00–317.25)	0.598	−0.03 (−0.15 to 0.08), negligible
MPV, fL (n = 407)	9.90 (9.15–10.60)	9.70 (9.10–10.55)	10.10 (9.30–10.70)	0.010	0.16 (0.04 to 0.27), small
NLR (n = 405)	2.03 (1.59–2.70)	1.95 (1.49–2.52)	2.20 (1.75–2.88)	0.001	0.20 (0.08 to 0.31), small
PLR (n = 405)	122.92 (100.54–155.31)	120.91 (100.80–156.50)	126.68 (99.74–154.13)	0.743	0.02 (−0.10 to 0.14), negligible
MLR (n = 405)	0.20 (0.16–0.25)	0.19 (0.16–0.24)	0.21 (0.16–0.26)	0.020	0.14 (0.03 to 0.26), small
SII (n = 405)	550.59 (392.97–762.82)	529.41 (375.68–729.50)	584.78 (437.46–835.65)	0.006	0.17 (0.05 to 0.28), small
SIRI (n = 405)	0.87 (0.61–1.23)	0.84 (0.57–1.13)	1.02 (0.71–1.44)	<0.001	0.24 (0.13 to 0.35), small
PIV (n = 405)	230.35 (157.59–361.33)	217.92 (146.27–336.27)	272.24 (185.62–415.28)	0.001	0.19 (0.08 to 0.31), small
Postmenopausal status	168 (41.2)	71 (26.1)	97 (71.3)	<0.001	OR 7.04 (4.46 to 11.13)
Diabetes mellitus	62 (15.2)	17 (6.2)	45 (33.1)	<0.001	OR 7.42 (4.07 to 13.53)
Hypertension	85 (20.8)	22 (8.1)	63 (46.3)	<0.001	OR 9.81 (5.68 to 16.94)
Nulliparity	21 (5.1)	11 (4.0)	10 (7.4)	0.161	OR 1.88 (0.79 to 4.46)
Hysteroscopy-guided D&C	5 (1.2)	4 (1.5)	1 (0.7)	0.669	OR 0.50 (0.08 to 3.19)

**Table 2:** Distribution of histopathological diagnoses (n = 408).

Histopathological Diagnosis	n	%
Normal, inactive or atrophic endometrium	106	26.0
Endometrial polyp	101	24.8
Proliferative endometrium	27	6.6
Secretory endometrium	22	5.4
Hyperplasia without atypia	13	3.2
Chronic endometritis	3	0.7
Benign, total	272	66.7
Endometrial intraepithelial neoplasia/atypical hyperplasia	43	10.5
Endometrial carcinoma or other uterine malignancy	93	22.8
Premalignant/malignant, total	136	33.3

Among the 93 carcinomas, the subtype was recorded in 74 (endometrioid: 68, serous: 5, carcinosarcoma: 1) and the grade in 72 (G1: 21, G2: 33, G3: 18).

### 3.2. Baseline Comparison

Cases were fourteen years older than controls (rank-biserial r = 0.58, 95% CI 0.49–0.68; large) and had thicker endometrium (r = 0.50, 0.40–0.60; large), and postmenopausal status, diabetes and hypertension were all far more common among them (all *p* < 0.001; Table 1). Differences in the indices were small by comparison: SIRI separated the groups best (r = 0.24, 0.13–0.35; small), followed by NLR and PIV (r = 0.20 and 0.19; both small), whereas PLR did not differ (r = 0.02, −0.10 to 0.14; negligible), nor did RDW, haemoglobin, platelet or lymphocyte counts (all |r| ≤ 0.06; negligible). Of the twenty-one continuous variables in Table 1, only age and endometrial thickness reached a large effect size, and only body mass index a moderate one; every haemogram-derived index fell in the small or negligible band. The blood count-to-curettage interval was longer in cases (median 7.5 versus 2 days; *p* = 0.005; r = 0.17, small).

### 3.3. Discrimination

Age (AUC 0.792, 95% CI 0.744–0.841) and endometrial thickness (AUC 0.749, 95% CI 0.697–0.800) discriminated substantially better than any haemogram-derived measure (Table 3, Figure 2). Neither non-ratio haemogram parameter discriminated usefully: RDW gave an AUC of 0.529 (0.471–0.587; *p* = 0.334) and MPV 0.578 (0.520–0.636; *p* = 0.010). RDW and MPV are not members of the pre-specified index set and were examined exploratorily (Section 2.3); Figure 2a is restricted to that set and therefore shows PLR, which belongs to it, but not RDW, which does not. The diagnostic performance of RDW and MPV is reported in full in Table 3, and the two non-significant results—PLR within the index set and RDW outside it—are stated in the text above.

**Figure 2:**
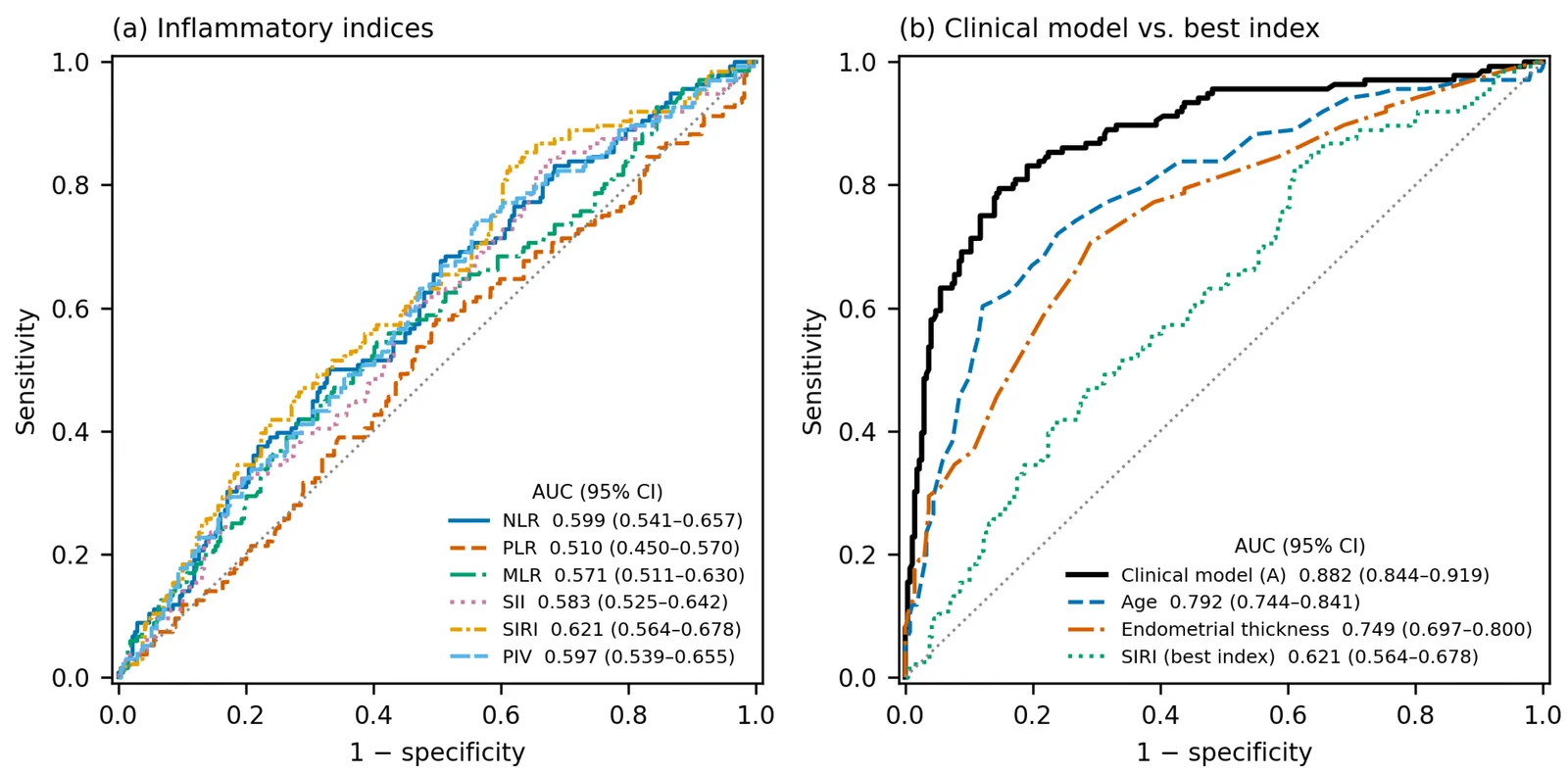
Receiver operating characteristic curves for (**a**) the six haemogram-derived inflammatory indices that constitute the pre-specified index set (RDW and MPV, examined exploratorily as non-ratio haemogram parameters, are not members of this set and are therefore not plotted; their AUCs are given in Table 3) and (**b**) the clinical model compared with its two strongest single components and the best-performing index. Areas under the curve are shown with DeLong 95% confidence intervals. SIRI was the best index (0.621, 0.564–0.678) and the remaining four non-PLR indices fell between 0.571 and 0.599; PLR did not discriminate (0.510, 0.450–0.570).

**Table 3:** Diagnostic performance of individual predictors. Cut-offs were derived by the Youden index in the same sample and are therefore optimistic. AUC confidence intervals are DeLong intervals; sensitivity and specificity intervals are Wilson intervals.

Predictor	n	AUC (95% CI)	*p*	Youden Cut-Off	Sensitivity, % (95% CI)	Specificity, % (95% CI)	LR+	LR−
Age	408	0.792 (0.744–0.841)	<0.001	58.0	60.3 (51.9–68.1)	87.9 (83.5–91.2)	4.97	0.45
Endometrial thickness	408	0.749 (0.697–0.800)	<0.001	11.0	70.6 (62.4–77.6)	71.0 (65.3–76.0)	2.43	0.41
NLR	405	0.599 (0.541–0.657)	0.001	1.91	67.6 (59.4–74.9)	49.4 (43.5–55.4)	1.34	0.65
PLR	405	0.510 (0.450–0.570)	0.743	121.43	58.1 (49.7–66.0)	50.2 (44.3–56.1)	1.17	0.84
MLR	405	0.571 (0.511–0.630)	0.020	0.21	54.4 (46.0–62.5)	59.5 (53.5–65.2)	1.34	0.77
SII	405	0.583 (0.525–0.642)	0.006	411.62	83.1 (75.9–88.5)	33.8 (28.4–39.7)	1.26	0.50
SIRI	405	0.621 (0.564–0.678)	<0.001	0.66	83.1 (75.9–88.5)	38.7 (33.0–44.6)	1.35	0.44
PIV	405	0.597 (0.539–0.655)	0.001	193.03	74.3 (66.3–80.9)	43.9 (38.1–49.8)	1.32	0.59
RDW	407	0.529 (0.471–0.587)	0.334	13.5	66.9 (58.6–74.3)	43.5 (37.8–49.5)	1.19	0.76
MPV	407	0.578 (0.520–0.636)	0.010	9.8	64.0 (55.6–71.6)	51.3 (45.4–57.2)	1.31	0.70

Fifteen pairwise DeLong comparisons were made (Supplementary Table S4). Uncorrected, six reached nominal significance: PLR was inferior to SIRI (ΔAUC −0.111, 95% CI −0.179 to −0.043; *p* = 0.001) and to NLR, PIV, SII and MLR (*p* = 0.002 to 0.042), and SIRI exceeded MLR (0.050; *p* = 0.018). After Bonferroni correction for fifteen comparisons (α = 0.0033), only the four differences involving PLR survived, and the SIRI–MLR difference did not. The remaining nine non-PLR pairs were indistinguishable throughout (all *p* ≥ 0.061). Pairwise Spearman correlations of 0.35 to 0.91 reflect the shared construction of the indices (Supplementary Table S5).

### 3.4. Incremental Value (Primary Analysis)

In univariable logistic regression, every index except PLR was associated with the outcome (Table 4).

**Table 4:** Univariable logistic regression for premalignant or malignant endometrial pathology. Odds ratios are maximum-likelihood estimates with Wald confidence intervals.

Predictor	n	Crude OR (95% CI)	*p*
Age, per 10 years	408	2.89 (2.27–3.68)	<0.001
Postmenopausal status	408	7.04 (4.45–11.15)	<0.001
Endometrial thickness, per 5 mm	408	2.22 (1.79–2.74)	<0.001
Diabetes mellitus	408	7.42 (4.04–13.61)	<0.001
Hypertension	408	9.81 (5.65–17.01)	<0.001
Nulliparity	408	1.88 (0.78–4.55)	0.160
Body mass index, per 5 kg/m^2^	191	1.61 (1.27–2.04)	<0.001
ln(NLR)	405	2.17 (1.35–3.50)	0.001
ln(PLR)	405	1.02 (0.56–1.85)	0.955
ln(MLR)	405	2.05 (1.14–3.69)	0.016
ln(SII)	405	1.70 (1.13–2.56)	0.012
ln(SIRI)	405	2.00 (1.37–2.90)	<0.001
ln(PIV)	405	1.62 (1.18–2.23)	0.003
RDW, per 1%	407	0.96 (0.88–1.06)	0.450
MPV, per 1 fL	407	1.22 (1.02–1.46)	0.026
CBC-to-curettage interval, per 7 days	408	1.23 (1.07–1.41)	0.003

The clinical model achieved a C-statistic of 0.882 (95% CI 0.844–0.919), optimism-corrected 0.876, calibration slope 0.963, with all five components contributing independently (Table 5).

**Table 5:** Components of the clinical reference model (Model A; n = 408). C-statistic 0.882 (95% CI 0.844–0.919); optimism-corrected 0.876; bootstrap-corrected calibration slope 0.963; bootstrap-corrected calibration intercept 0.000; apparent Brier score 0.122; Nagelkerke R^2^ 0.529; Hosmer–Lemeshow *p* = 0.500. β is the log-odds regression coefficient with its standard error; the intercept is reported so that the model equation is complete and externally applicable. Odds ratios are from multivariable logistic regression with Wald confidence intervals and may differ marginally from the unadjusted odds ratios in Table 1.

Variable	β (SE)	Adjusted OR (95% CI)	*p*
Intercept	−6.0630 (0.9285)	—	—
Age, per 10 years	+0.4514 (0.1839)	1.57 (1.10–2.25)	0.014
Postmenopausal status	+1.0461 (0.3860)	2.85 (1.34–6.07)	0.007
Endometrial thickness, per 5 mm	+0.8990 (0.1343)	2.46 (1.89–3.20)	<0.001
Diabetes mellitus	+0.9502 (0.4154)	2.59 (1.15–5.84)	0.022
Hypertension	+1.0957 (0.3661)	2.99 (1.46–6.13)	0.003

The complete equation of the clinical model is given in Table 5. The linear predictor is LP = −6.063 + 0.451 × (age in years/10) + 1.046 × (postmenopausal) + 0.899 × (endometrial thickness in mm/5) + 0.950 × (diabetes mellitus) + 1.096 × (hypertension), with the binary terms coded 1 if present and 0 if absent, and the predicted probability is 1/(1 + e^(^^−LP)^). Calibration was assessed at two levels. The apparent calibration slope is 1.000 by construction; the bootstrap-corrected slope was 0.963 and the cross-validated slope 0.942. The apparent calibration intercept was 0.000, the bootstrap-corrected intercept 0.000 and the cross-validated intercept +0.002, and the Brier score was 0.122 apparent and 0.126 after correction.

These calibration quantities are internal to the sampled data and must not be read as calibration-in-the-large for any clinical population. Because cases and controls were sampled at a fixed 1:2 ratio, the intercept is determined by the sampling fraction rather than by the underlying prevalence, so mean calibration is not identifiable from this design [64]; this is the same constraint that makes absolute risk and net benefit unavailable here (Section 2.5). For application in a population with prevalence π, the intercept requires the standard case–control correction β0(π) = −6.063 − ln[(136/272)/(π/(1 − π))], which gives −9.262, −8.314 and −7.461 at prevalences of 2%, 5% and 11%, respectively—the three prevalences at which predictive values are projected in Supplementary Table S3. The calibration slope and the five covariate coefficients are unaffected by the sampling ratio and transfer unchanged.

Against this model, no index retained significance, and none approached the 0.02 threshold for clinical relevance (Table 6, Figure 3).

**Table 6:** Primary analysis: incremental value of each index over the clinical model, and the diagnostic performance of every model. Refitted in the identical n = 405 subset (136 cases, 269 controls), Model A gives a C-statistic of 0.882 (95% CI 0.845–0.920), the reference from which every ΔC is computed. The likelihood-ratio test is the primary inferential test; the DeLong *p*-value is given for comparability with prior literature only and is conservative for nested models [55]. The Bonferroni threshold for the six tests is *p* < 0.0083. Panel A gives the inferential comparison; Model A is shown as the reference row, and Model H, whose four estimable terms are given in Table 7, is shown for completeness. Panel B gives the diagnostic measures. In Panel B, the cut-off is the value of the predicted probability that maximises the Youden index, derived in the same sample and therefore optimistic, exactly as for the single predictors in Table 3; confidence intervals are Wilson intervals; optimism-corrected accuracy is the apparent value less the bootstrap estimate of optimism over 500 replicates in which both the model and its cut-off were re-estimated. Because the cut-off differs between models, the sensitivity–specificity pairs do not lie at a common operating point; the final column therefore evaluates every model at the single cut-off of the clinical model (0.366) and compares its classification with that of the clinical model in the same women by McNemar’s test. Model C classified all 405 women identically to Model A at that cut-off, so the test is undefined there. Predicted probabilities lie on the scale of a 1:2 case–control sample and accuracy is a function of prevalence, so neither is transferable to a population with a different prevalence (Section 3.4).

**Panel A. Incremental Value of Each Index over the Clinical Model.**							
**Model**	**n**	**Adjusted OR per log unit (95% CI)**	***p*** **(Wald)**	**LR χ^2^ (df)**	***p*** **(LR)**	**ΔC (95% CI)**	***p*** **(DeLong)**
Model A = clinical model (reference)	405	—	—	—	—	0 (reference)	—
Model B = A + ln(NLR)	405	1.61 (0.85–3.06)	0.143	2.17 (1)	0.141	+0.0042 (−0.0027 to +0.0111)	0.236
Model C = A + ln(PLR)	405	0.90 (0.40–2.03)	0.796	0.07 (1)	0.796	−0.0004 (−0.0018 to +0.0010)	0.546
Model D = A + ln(MLR)	405	1.35 (0.61–2.96)	0.460	0.55 (1)	0.459	+0.0010 (−0.0020 to +0.0040)	0.521
Model E = A + ln(SII)	405	1.23 (0.71–2.14)	0.467	0.53 (1)	0.467	+0.0014 (−0.0021 to +0.0048)	0.438
Model F = A + ln(SIRI)	405	1.37 (0.84–2.25)	0.210	1.59 (1)	0.207	+0.0028 (−0.0022 to +0.0078)	0.273
Model G = A + ln(PIV)	405	1.16 (0.76–1.77)	0.505	0.45 (1)	0.504	+0.0010 (−0.0017 to +0.0038)	0.466
Model H = A + four-index basis	405	Four terms; see Table 7	—	4.48 (4)	0.345	+0.0064 (−0.0025 to +0.0152)	0.158
**Panel B. Diagnostic Measures for Each Model.**							
**Model**	**Youden cut-off**	**Sensitivity, % (95% CI)**	**Specificity, % (95% CI)**	**Accuracy, % (95% CI)**		**Accuracy, optimism-corrected, %**	**Accuracy at common cut-off, % (McNemar** ***p*** **)**
Model A = clinical model (reference)	0.366	77.9 (70.3–84.1)	86.2 (81.6–89.9)	83.5 (79.5–86.8)		82.2	83.5 (reference)
Model B = A + ln(NLR)	0.259	87.5 (80.9–92.0)	79.2 (73.9–83.6)	82.0 (77.9–85.4)		80.6	83.5 (1.000)
Model C = A + ln(PLR)	0.354	78.7 (71.1–84.7)	85.9 (81.2–89.5)	83.5 (79.5–86.8)		82.1	83.5 (—)
Model D = A + ln(MLR)	0.249	86.8 (80.0–91.5)	79.6 (74.3–83.9)	82.0 (77.9–85.4)		80.6	83.0 (0.688)
Model E = A + ln(SII)	0.256	86.8 (80.0–91.5)	79.6 (74.3–83.9)	82.0 (77.9–85.4)		80.6	83.7 (1.000)
Model F = A + ln(SIRI)	0.262	86.8 (80.0–91.5)	79.9 (74.7–84.3)	82.2 (78.2–85.6)		80.8	83.0 (0.727)
Model G = A + ln(PIV)	0.251	86.8 (80.0–91.5)	79.2 (73.9–83.6)	81.7 (77.7–85.2)		80.3	83.2 (1.000)
Model H = A + four-index basis	0.295	84.6 (77.5–89.7)	84.0 (79.2–87.9)	84.2 (80.3–87.4)		82.4	83.2 (1.000)

**Table 7:** Joint model (Model H = Model A + the identifiable four-index basis; n = 405). C-statistic 0.888 (apparent), against 0.882 for Model A in the same 405 women; likelihood-ratio χ^2^_4_ = 4.48, *p* = 0.345. The six log-indices span a four-dimensional space, so a model containing all six is rank-deficient and its individual coefficients are not identifiable; ln(SII) and ln(PIV) are exact linear combinations of the four terms shown and add no information. Odds ratios are per one-unit increase in the natural logarithm of the index. VIF, variance inflation factor.

Term	β	Adjusted OR (95% CI)	*p*	VIF
Intercept	−2.2270	—	—	—
Age, per 10 years	+0.4337	1.54 (1.07–2.22)	0.020	2.53
Postmenopausal status	+1.0936	2.99 (1.38–6.45)	0.005	2.19
Endometrial thickness, per 5 mm	+0.8641	2.37 (1.80–3.13)	<0.001	1.13
Diabetes mellitus	+0.9306	2.54 (1.10–5.84)	0.029	1.38
Hypertension	+1.1430	3.14 (1.51–6.51)	0.002	1.53
ln(NLR)	+1.1476	3.15 (0.66–15.00)	0.149	6.10
ln(PLR)	−0.8837	0.41 (0.12–1.42)	0.161	2.35
ln(MLR)	+0.1873	1.21 (0.24–6.16)	0.822	4.34
ln(SIRI)	−0.3235	0.72 (0.18–2.90)	0.648	8.04
ln(SII)	not identifiable	=ln(PLR) + ln(SIRI) − ln(MLR)	—	—
ln(PIV)	not identifiable	=ln(SII) + ln(SIRI) − ln(NLR)	—	—

**Figure 3:**
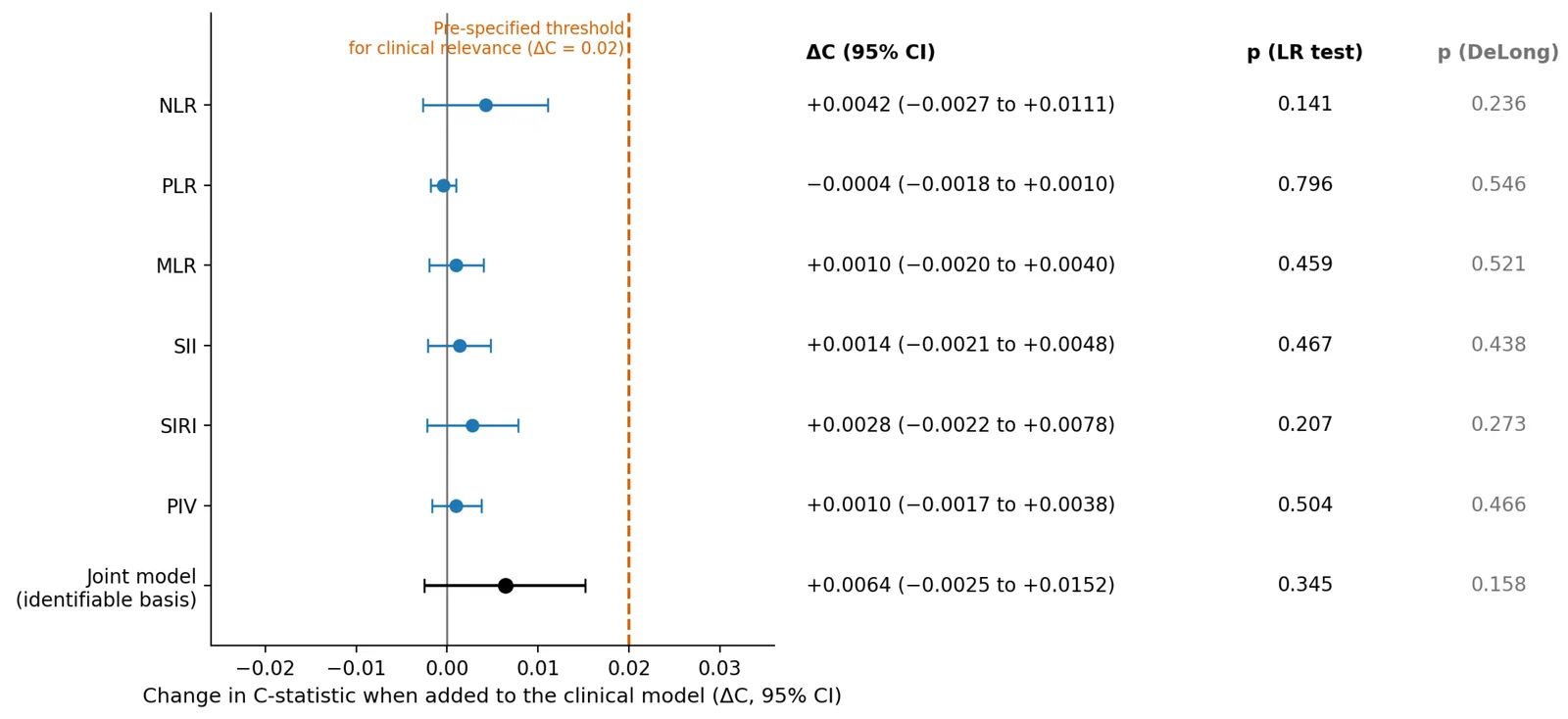
Change in C-statistic (ΔC) when each log-transformed index, and all six jointly, are added to the clinical model. Error bars are DeLong 95% confidence intervals. Two *p*-values are shown: the likelihood-ratio test, which is the primary inferential test, and the DeLong test, reported for comparability with prior literature and conservative for nested models [55]. The dashed line marks the ΔC of 0.02 adopted as the threshold for clinical relevance. On the likelihood-ratio test, the primary inferential test, no index term reached significance: the smallest *p*-value was 0.141 for NLR and the next 0.207 for SIRI, neither approaching the Bonferroni threshold of 0.0083. Wald tests agreed closely (0.143 and 0.210), whereas DeLong gave systematically larger values (0.236 and 0.273), as expected for a test that is conservative under nesting [55]. The largest gain in discrimination was ΔC = +0.0042 (−0.0027 to +0.0111) for NLR, an order of magnitude below the 0.02 threshold; SIRI gave +0.0028, and PLR contributed nothing. Entered jointly, the indices raised the C-statistic from 0.882 to 0.888 (ΔC = +0.0064, −0.0025 to +0.0152). Because two exact algebraic identities link the six log-indices, the joint model is fitted with a maximal identifiable basis of four of them (Section 2.5); this reproduces the C-statistic and the likelihood-ratio statistic of the six-term specification exactly, and the test carries four rather than six degrees of freedom (χ^2^_4_ = 4.48; *p* = 0.345). The increment did not survive internal validation (Table 8).

**Table 8:** Internal validation of the clinical model and of the joint model. Optimism was estimated by bootstrap resampling with 500 replications; cross-validation used 10 folds repeated 20 times, with performance recomputed on the pooled out-of-fold linear predictors. The calibration intercept is reported for completeness only: under a fixed 1:2 case–control sampling ratio it reflects the sampling fraction and is not calibration-in-the-large for any clinical population (Section 3.4). Model A is shown both in the full cohort and in the 405-woman analytic subset in which every index was computable, so that the two models are compared in identical women.

Model	n	Validation	C-Statistic	Calibration Slope	Calibration Intercept	Brier Score
Model A (clinical)	408	Apparent	0.882	1.000	0.000	0.122
	408	Optimism-corrected	0.876	0.963	0.000	0.126
	408	Cross-validated	0.874	0.942	+0.002	0.126
Model A (clinical)	405	Apparent	0.882	1.000	0.000	0.122
	405	Optimism-corrected	0.876	0.958	−0.007	0.127
	405	Cross-validated	0.874	0.938	+0.001	0.126
Model H (A + indices)	405	Apparent	0.888	1.000	0.000	0.119
	405	Optimism-corrected	0.876	0.919	−0.006	0.127
	405	Cross-validated	0.875	0.901	+0.003	0.127
ΔC, Model H − Model A	405	Apparent	+0.0064			
	405	Optimism-corrected	−0.0001			
	405	Cross-validated	+0.0010 (−0.0076 to +0.0097); *p* = 0.814			

Diagnostic measures for every model are given in Panel B of Table 6. At its own Youden cut-off, the clinical model classified with a sensitivity of 77.9% (95% CI 70.3–84.1), a specificity of 86.2% (81.6–89.9) and an accuracy of 83.5% (79.5–86.8). Adding an index moved the operating point rather than improving the classification. For five of the six indices, the cut-off fell from 0.366 to between 0.249 and 0.262, sensitivity rose to 86.8–87.5% and specificity fell to 79.2–79.9%, so that accuracy was 81.7% to 82.2% in every case below the 83.5% of the clinical model alone. PLR, the one index that did not discriminate univariably, left the cut-off and every measure essentially unchanged (accuracy 83.5%). The joint model gave the highest accuracy, 84.2% (80.3–87.4), which is three more women correctly classified out of 405 than by the clinical model. Bootstrap optimism correction lowered every estimate by 1.2 to 1.8 percentage points and left the clinical model above every single-index model (clinical model 82.2%; single-index models 80.3% to 82.1%; joint model 82.4%).

Because these cut-offs differ between models, the sensitivity–specificity pairs above do not lie at a common operating point and are not directly comparable with one another. Evaluated instead at the single cut-off of the clinical model, all eight models classified these women almost identically: accuracy ranged from 83.0% to 83.7% against 83.5% for the clinical model, the largest difference in either direction being two women in 405, and no comparison approached significance on McNemar’s test (smallest *p* = 0.688; Model C classified every woman identically to Model A, so the test is undefined there). The diagnostic measures therefore carry the same conclusion as the likelihood-ratio tests and the ΔC estimates: none of the six indices alters the classification of these women to a degree capable of affecting a triage decision.

Entering the indices jointly requires care, because the six log-indices are not linearly independent. They are multiplicative functions of only four cell counts and satisfy two exact identities, ln(SII) = ln(PLR) + ln(SIRI) − ln(MLR) and ln(PIV) = ln(SII) + ln(SIRI) − ln(NLR); the six-column design matrix therefore has rank four, with two singular values of exactly zero, and coefficients for all six indices are not estimable. The joint model (Model H) was accordingly fitted with the maximal identifiable basis of four log-indices—NLR, PLR, MLR and SIRI—which reproduces the fitted values, the C-statistic (0.888) and the likelihood-ratio statistic (χ^2^ = 4.48) of the six-term specification exactly, on four rather than six degrees of freedom (*p* = 0.345). Within that model, no individual index contributed independently (Table 7): the smallest *p*-value was 0.149 for ln(NLR), and variance inflation factors of 2.35 to 8.04 show that substantial shared information remains even after the exact dependencies have been removed. The five clinical covariates retained their independent contributions essentially unchanged, with adjusted odds ratios of 1.54 to 3.14.

Because an apparent gain of this size in a ten-parameter model is precisely the situation in which optimism matters, the comparison was repeated under internal validation (Table 8). The apparent increment of +0.0064 was optimism in its entirety: the bootstrap-corrected C-statistic of the joint model was 0.876, identical to that of the clinical model (corrected ΔC = −0.0001), and under 10-fold cross-validation repeated 20 times, the difference was +0.0010 (95% CI −0.0076 to +0.0097; DeLong *p* = 0.814). Calibration deteriorated when the indices were added, with the bootstrap-corrected calibration slope falling from 0.958 to 0.919 and the cross-validated slope from 0.938 to 0.901—both within the 405-woman subset in which the two models are compared, where the corrected slope of Model A is 0.958 rather than the 0.963 obtained in the full cohort—and the corrected Brier score did not improve (0.127 in both models). The upper confidence limit of the cross-validated increment, +0.0097, lies below the 0.02 threshold, so the conclusion drawn from the apparent analysis is not merely preserved but strengthened by validation.

Mean platelet volume, examined exploratorily as a non-ratio parameter, was the only haemogram measure nominally associated with the outcome after adjustment (OR 1.40 per fL, 1.09–1.79; *p* = 0.0074), but it did not survive correction for the seven haemogram tests (threshold *p* < 0.0071) and its ΔC was +0.0074 (−0.0014 to +0.0163; *p* = 0.099).

The precision of these estimates defines what the study can and cannot exclude. The upper 95% confidence bound on ΔC was +0.0111 for the best single index and +0.0152 for all six entered jointly; under cross-validation, the upper bound for the joint model fell to +0.0097. The data are therefore incompatible with an incremental gain approaching the 0.02 threshold but remain compatible with gains of up to roughly 0.015, which this sample is not large enough to rule out.

### 3.5. Stratified, Interaction and Sensitivity Analyses

No interaction between menopausal status and any index approached significance (smallest *p* = 0.143). AUCs were numerically higher among postmenopausal women (SIRI 0.664 versus 0.603), but the clinical model also retained most of its discrimination within strata (C = 0.862 and 0.803) and incremental value remained negligible in both (SIRI ΔC = +0.0032 and +0.0035; Supplementary Table S6).

The direction of the result was consistent across all twelve sensitivity analyses; no index was significant in any of them except in the body mass index sub-cohort, which is examined below (Supplementary Table S7, which gives n and event counts). Restricting cases to carcinoma alone removed every association (SA1; adjusted ORs 0.94–1.22; all *p* ≥ 0.695), and excluding hysteroscopy-guided procedures (SA2), the 17 repaired records (SA3), adjusting for the blood count-to-curettage interval (SA4), trimming the extreme 1% of each index (SA7) and penalised regression (SA8) all gave ΔC below 0.005. Setting to missing the indices of the three records that failed the internal-consistency rule but were retained as entered (SA12; n = 402, 135 events) left every estimate essentially unchanged: adjusted odds ratios ran from 0.90 to 1.64, the smallest likelihood-ratio *p*-value was 0.130 for NLR, and the largest ΔC was +0.0045, again for NLR.

Two analyses are exceptions to the claim that 0.02 is excluded and are named as such. Restricting cases to 2025–2026 (SA5; n = 323, 54 events) gave a SIRI upper bound of +0.0229. The body mass index sub-cohort (SA6; n = 191, 72 events) gave upper bounds of +0.0376 for NLR and +0.0369 for SIRI and is the only analysis in which any index term reached nominal significance (NLR OR 3.51, 1.29–9.52, *p* = 0.014; SIRI 3.23, 1.42–7.34, *p* = 0.005). Two checks located the source of that signal. Removing BMI from the model within the same sub-cohort barely changed the estimates (NLR 3.32, *p* = 0.016; SIRI 2.90, *p* = 0.008), so it belongs to the sub-cohort rather than to adjustment for obesity; and women with a recorded BMI had higher NLR (median 2.08 versus 1.99; *p* = 0.010) and thicker endometrium (10 versus 9 mm; *p* = 0.037) than those without, while age, menopausal status and comorbidity did not differ (SA11; Supplementary Table S7b). In both SA5 and SA6, the point estimates for ΔC remain far below 0.02, but the intervals are too wide to exclude it; both analyses are substantially smaller than the primary one, and the SA6 finding is reported as hypothesis-generating.

The drift probe was unable to detect a change in any index or cell count across 2021–2026 after Benjamini–Hochberg correction, but it is low-powered: with 136 cases it has about 80% power for a correlation of 0.24, so smaller drift cannot be excluded. The largest raw correlation, for the monocyte count, was rho = +0.205 (+0.038 to +0.361; uncorrected *p* = 0.017, adjusted *p* = 0.187), in the direction that would matter (Supplementary Table S9). Finally, data repair affected the result and is reported explicitly: before the internal-consistency rule was applied, SIRI appeared to be an independent predictor (adjusted OR 1.74, 1.14–2.64; *p* = 0.010), and after the thirteen records with an identifiable error were repaired or set to missing, the same estimate became 1.37 (0.84–2.25; *p* = 0.210). The null does not, however, rest on those repair decisions: excluding every record touched by repair (SA3) and setting the three retained records to missing (SA12) both leave the adjusted estimate non-significant.

## 4. Discussion

In 408 women undergoing dilatation and curettage for abnormal uterine bleeding, six haemogram-derived inflammatory indices showed weak univariable associations with premalignant or malignant endometrial pathology, SIRI discriminating best at an AUC of 0.621 and PLR not at all. Against a clinical model containing age, menopausal status, endometrial thickness, diabetes and hypertension—which reached a C-statistic of 0.882 in this sample, optimism-corrected 0.876, with a calibration slope of 0.963—no index produced a detectable gain in discrimination. The apparent gain of +0.0064 obtained when the indices were entered jointly was optimism in its entirety: it fell to −0.0001 after bootstrap correction and to +0.0010 (−0.0076 to +0.0097) under repeated cross-validation, while calibration deteriorated. Expressing performance as sensitivity, specificity and accuracy rather than as a C-statistic did not change that conclusion: evaluated at a common operating point, every index model classified these women within 0.5 percentage points of the clinical model, and no comparison approached significance.

That figure is not the performance of a triage model in clinical use. It is the discrimination of these covariates in a sample where prevalence is fixed at 33.3% by design and the age distributions barely overlap; restricting to the shared age range already lowers it to 0.856, and in an unselected series it would fall further. The consequence for the incremental question is uncertain rather than reassuring: a weaker comparator would leave more room for an index, but the indices are inflated by the same mechanism, so the net direction of the bias on ΔC cannot be determined here. The largest single-index gain was 0.0042, and all six together added 0.0064, neither approaching the 0.02 threshold.

What follows is bounded by the precision of these estimates. With an upper confidence limit of 0.0111 for the best single index and 0.0152 for the joint model, falling to 0.0097 for the joint model under cross-validation, the study excludes a gain of the size that would matter clinically but not a small one, and supports the conclusion that the indices are not ready for pre-curettage triage rather than that they carry no information.

Two mechanisms could explain the loss of the crude associations: confounding by the clinical profile, or information that is genuine but redundant. The pattern fits the first, though the two are not fully separable in observational data. Women with neoplasia here were fourteen years older and three, five and six times as likely to be postmenopausal, diabetic and hypertensive, respectively, each independently associated with a mildly raised index; and adjusted odds ratios shifted towards unity with intervals spanning it symmetrically.

Our result appears to conflict with diagnostic series reporting differences between pathology groups, and the conflict is instructive: those studies report that indices differ between groups, and so do we—five of six differed univariably. Where we differ is in what the comparison is against. Qin reported an NLR AUC of 0.777 in 80 cancers versus 74 benign uterine diseases [37]; Ronsini et al. reported 0.635 for SII multiplied by endometrial thickness [42], close to ours and obtained by incorporating the very clinical variable we show dominates; Kumari et al. reported differences on only 11 carcinomas [38]. Recent series follow the same pattern: Xu et al. report an AUC of 0.936, but from a model combining body mass index, CA125, HE4, albumin and PLR, so the inflammatory contribution is not separable [43]; a machine learning model reports high accuracy without a clinical comparator [44]; and a 2026 series again reports index differences without asking what they add to age and endometrial thickness [45]. None compared its index with a clinical model. When that comparison is made, the contribution is no longer detectable at the precision available here.

Three findings deserve comment. PLR did not discriminate, contradicting Acmaz et al., in whom PLR separated both hyperplasia and carcinoma from normal endometrium while NLR separated only carcinoma from controls [35], and agreeing with Ilgen et al., in whom NLR discriminated and PLR did not [36]; that two studies in the same population disagree about which of two highly correlated ratios matters suggests neither carries a robust signal. Restricting cases to carcinoma removed every association, which fits the indices tracking the phenotype of the women rather than the lesion. Mean platelet volume, the only measure nominally significant after adjustment, we report but do not claim: it failed correction for multiplicity, its ΔC was 0.0074, and it lay outside the pre-specified index set.

The study has methodological strengths. All six indices were assessed simultaneously in the same women, allowing head-to-head comparison rather than indirect comparison across studies, and all were tested for incremental value against a comparator fixed on clinical rather than statistical grounds. Indices were recalculated from raw differential counts, and a written data-quality rule set detected sixteen records failing an internal-consistency check, thirteen of them with identifiable entry errors that would otherwise have propagated into every ratio—a step rarely reported here, and one that changed the result, since the only index significant before repair was not significant after it. The full log of plan revisions, including which decisions followed the first look at the data, is published with the paper rather than left implicit. Two further methodological points strengthen the analysis. The exact algebraic dependencies among the six log-indices were identified and handled explicitly, so the joint model is identifiable and its likelihood-ratio test carries the correct four degrees of freedom rather than the six a naive specification would assume. And the incremental comparison was not left at the apparent level: bootstrap optimism correction and repeated cross-validation both removed the small apparent gain, and calibration as well as discrimination was reported at each stage.

The limitations are substantial. Cases were ascertained across 2021 to 2026 whereas all controls came from the first half of 2026, so measurement period and group membership are partly confounded. Three observations bear on this: the drift probe detected no change after correction for multiplicity, though it is low-powered; restricting cases to 2025–2026 and, separately, excluding the 33 pandemic-period cases left the result unchanged; and every patient operated during the pandemic had a documented negative preoperative SARS-CoV-2 PCR test, so active infection at sampling is excluded, although this does not exclude residual effects of past infection or vaccination. The limitation of this defence should be stated plainly: what would settle the question is not drift within cases but an interaction between case–control status and calendar period, and with no control sampled before 2026 that interaction cannot be estimated at all.

Cases and controls were not matched on age or menopausal status, so the pooled contrast overstates what a clinician faces, and adjusting for a variable differing this much is itself a source of uncertainty. Stratum-specific results are reported alongside the pooled ones and the null holds in both, and restricting to the 45-to-65-year range shared by both groups likewise left every index non-significant. A diagnostic case–control design is generally expected to inflate apparent accuracy through spectrum effects, so the sampling should, if anything, have favoured the indices, though that argument is qualitative.

Body mass index was missing in 53% of women, so the strongest modifiable risk factor for endometrial cancer is unadjusted in the primary analysis. In the sub-cohort where it was recorded, NLR and SIRI were significant, and a sceptical reading would hold that our null reflects residual confounding by obesity. That reading can be tested rather than argued about. Removing BMI from the model within the same sub-cohort left the estimates almost unchanged, so the signal belongs to the sub-cohort and not to adjustment for obesity; women with a recorded BMI had higher NLR and thicker endometrium, identifying the sub-cohort as selected; and the point estimates of the gain there stayed below 0.013, although their intervals reach 0.038 and span zero. These observations make confounding by obesity an unlikely explanation without excluding it, and the finding warrants prospective testing where BMI is recorded for everyone.

The reference standard is imperfect, and its errors are unlikely to be random. Endometrial sampling misses about 10% of carcinomas and 18% of atypical hyperplasias when hysteroscopy is taken as the reference standard, and fewer when dilatation and curettage is [12], so these figures are the conservative bound; the control group must therefore contain some missed lesions, and because sampling failure concentrates in atrophic postmenopausal endometrium these fall preferentially in the stratum where the indices performed numerically best (SIRI 0.664 versus 0.603)—differential misclassification biased towards the null there. Its magnitude can be bounded: applying those sensitivities to a source prevalence of 2% to 11%, between about one and five of the 272 controls would be expected to harbour an undetected lesion, too few to generate a null of the size observed. A further 47 specimens (3.4% of the frame) were reported as insufficient and excluded before sampling; this is far below the 31% of the referral series [12], but it removes a typically atrophic, postmenopausal group and so narrows the spectrum of the control arm.

Tamoxifen and hormone replacement therapy were not retrievable and remain unmeasured confounders. The analysis plan is also not fully pre-specified: the primary endpoint, the ΔC threshold and the composition of the comparator model were fixed in protocol v3.0 after the results of the original plan had been seen, and five further groups of analyses were added during peer review (Section 2.1; Supplementary Table S1). The complete timing is published so that readers can weight the findings accordingly, and we note that choices made after the fact can manufacture a positive result far more readily than a null one: every analysis added after the results were known, including all four requested by the reviewers, moved the conclusion in the same direction. Finally, this is a single-centre study and absolute risks are not estimable from its design. For the same reason, the diagnostic measures in Panel B of Table 6 describe classification within a sample whose prevalence is fixed at 33.3% by the sampling ratio, and the cut-offs at which they were obtained were derived in that same sample; both would differ in an unselected population, which is why optimism-corrected accuracy is reported alongside the apparent value.

The clinical implication is narrow. This does not establish that systemic inflammation is irrelevant to endometrial carcinogenesis; the prognostic literature is substantial and biologically coherent. It suggests that, at the precision available here, measuring these ratios does not improve a triage decision already being made on age, menopausal status and endometrial thickness. A systematic review of nineteen published models for endometrial cancer risk found C-statistics from 0.56 to 0.92, most models at high risk of bias and only five reporting calibration [58], and models built from age, body mass index and endometrial thickness alone already discriminate well in this setting [73]; a candidate marker must therefore be judged against such a baseline rather than against nothing. Future work should compare candidate markers against that clinical baseline rather than against nothing and report incremental value rather than univariable association; prospective evaluation in an unselected consecutive cohort would be the appropriate next step.

## 5. Conclusions

In 408 women undergoing dilatation and curettage for abnormal uterine bleeding, none of six haemogram-derived inflammatory indices—NLR, PLR, MLR, SII, SIRI or PIV—added detectable discrimination to a model built from age, menopausal status, endometrial thickness, diabetes and hypertension. The clinical model alone achieved a C-statistic of 0.882 in this sample—a figure inflated by the case–control design and not an estimate of clinical performance—and the largest incremental gain from any index was 0.004, with an upper confidence limit of 0.011, well below the 0.02 threshold adopted as clinically relevant. Entering all six jointly raised the apparent C-statistic by 0.006, but this was optimism: the increment was −0.0001 after bootstrap correction and +0.0010 (upper limit +0.0097) under repeated cross-validation, and calibration worsened. The weak univariable associations seen for five of the six indices are most consistent with the older, more often postmenopausal and more metabolically comorbid profile of affected women rather than with an independent biological signal, and they were no longer present when cases were restricted to carcinoma. Based on this evidence, these indices are not ready to be adopted for pre-curettage triage in abnormal uterine bleeding, and univariable comparisons alone are not an adequate basis for doing so.

## Figures and Tables

**Figure 1:**
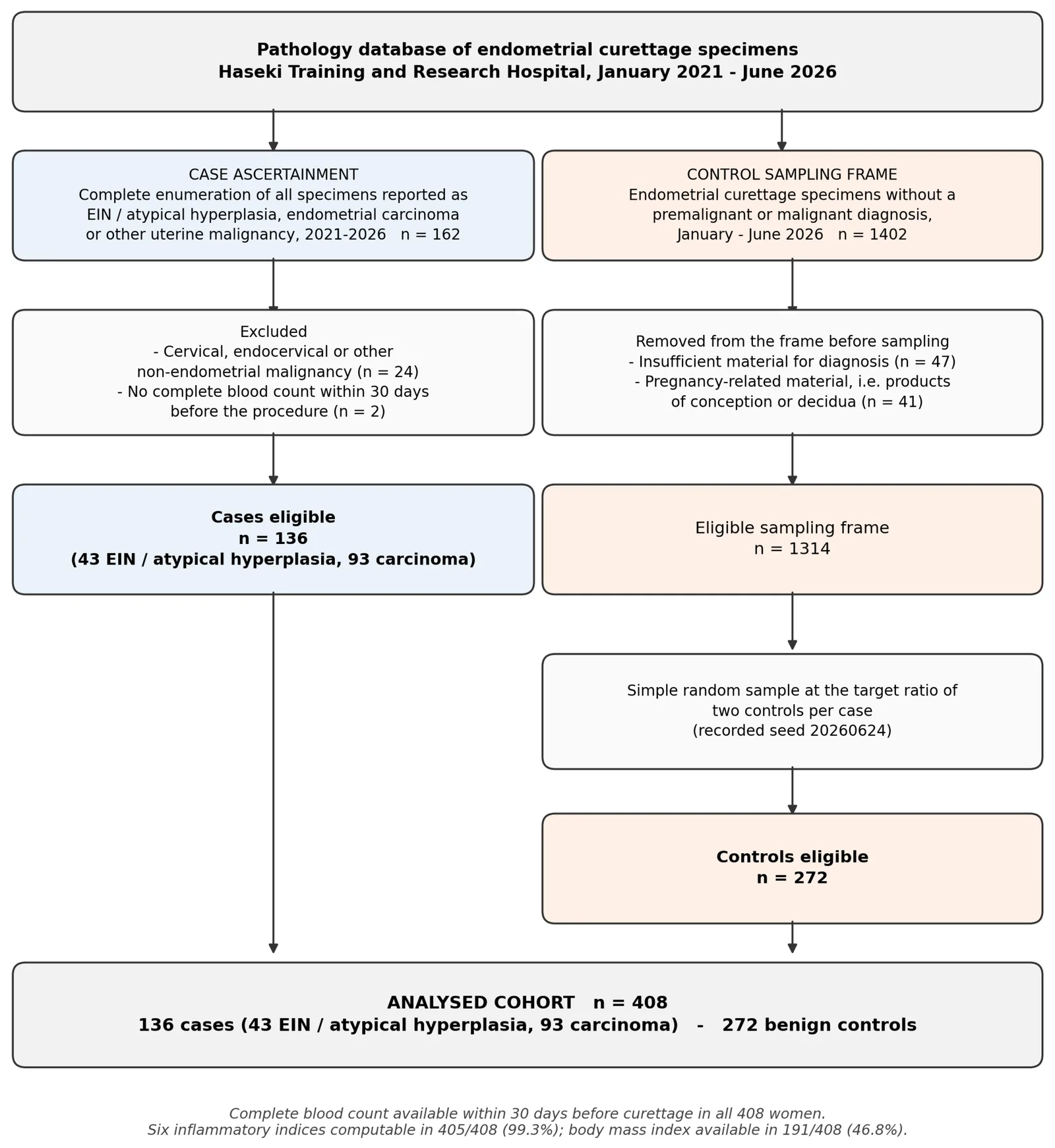
Patient flow. Cases were ascertained by complete enumeration of the 162 specimens reported as premalignant or malignant across 2021–2026; controls were drawn by reproducible random sampling at a fixed 1:2 ratio from an eligible frame of 1314 benign endometrial curettage specimens reported between January and June 2026.

## Data Availability

The individual-level dataset is not publicly available because it derives from hospital medical records and, in combination with age, calendar period of curettage and histopathological diagnosis, could permit re-identification of individual patients within the source institution. Anonymised individual-level data and the analysis code are available from the corresponding author on reasonable request, subject to approval by the institutional Clinical Research Ethics Committee of Haseki Training and Research Hospital.

## Supplementary Materials

The following supporting information can be downloaded at: https://www.mdpi.com/article/10.3390/diagnostics16193219/s1, Table S1. Protocol deviation log; Table S2. Data-cleaning rule set and record-level repair log; Table S3. Predictive values projected at realistic prevalences; Table S4. Pairwise DeLong comparisons among the six indices; Table S5. Spearman correlation matrix among the six indices; Table S6. Discrimination stratified by menopausal status; Table S7. Sensitivity analyses SA1 to SA10 and SA12; Table S7b. Sensitivity analysis SA11, comparing women with and without a recorded body mass index; Table S8. Missing data by source; Table S9. Temporal drift probe within the case group (2021–2026); Table S10. Chi-square and Fisher’s exact test results for all categorical variables; Figure S1. Box plots with individual observations for the six haemogram-derived inflammatory indices in the benign and premalignant/malignant groups (logarithmic y-axis). Boxes show the median and interquartile range; whiskers extend to 1.5 times the interquartile range. *P*-values are from the Mann–Whitney U test. The distributions overlap almost completely, which is the visual counterpart of the areas under the curve of 0.51 to 0.62 reported in Table 3.

## Abbreviations

The following abbreviations are used in this manuscript:

AUB	Abnormal uterine bleeding
AUC	Area under the receiver operating characteristic curve
BMI	Body mass index
CBC	Complete blood count
CI	Confidence interval
D&C	Dilatation and curettage
EIN	Endometrial intraepithelial neoplasia
IQR	Interquartile range
LR	Likelihood ratio (LR+, LR−) and likelihood-ratio test (LR χ^2^)
MCV	Mean corpuscular volume
MLR	Monocyte-to-lymphocyte ratio
MPV	Mean platelet volume
NLR	Neutrophil-to-lymphocyte ratio
OR	Odds ratio
PCR	Polymerase chain reaction
PIV	Pan-immune-inflammation value
PLR	Platelet-to-lymphocyte ratio
RDW	Red cell distribution width
SA	Sensitivity analysis (SA1–SA11)
SE	Standard error
SII	Systemic immune-inflammation index
SIRI	Systemic inflammation response index
STARD	Standards for Reporting of Diagnostic Accuracy Studies
STROBE	Strengthening the Reporting of Observational Studies in Epidemiology
TRIPOD	Transparent Reporting of a multivariable prediction model for Individual Prognosis Or Diagnosis
VIF	Variance inflation factor
ΔC	Change in the C-statistic
