# Supplementary material for: Limited Incremental Value of Six Haemogram-Derived Inflammatory Indices for Detecting Endometrial Carcinoma and Intraepithelial Neoplasia in Abnormal Uterine Bleeding: A Case–Control Study
Source: Diagnostics (Basel). 2026 Oct 5;16(19):3219. doi: 10.3390/diagnostics16193219 (PMC13664213; doi:10.3390/diagnostics16193219)
Supplement: Supplementary file 1 [file diagnostics-16-03219-s001.zip › diagnostics-4525229-supplementary.pdf]

Supplementary Materials

Limited Incremental Value of Six Haemogram-Derived Inflammatory Indices for Detecting Endometrial Carcinoma and Intraepithelial Neoplasia in Abnormal Uterine Bleeding: A Case–Control Study

Table S1. Protocol deviation log

#	Original protocol (v2.0)	Final protocol (v3.0)	Reason	When decided
1	"Retrospective cross-sectional"	Unmatched case–control (1:2)	The original descriptor misdescribed what was done; sampling was outcome-dependent. The conduct of the study did not change, only its label	Before/during data collection
2	Homogeneous 5-year period	Cases 2021–2026, controls January–June 2026	Source constraint; controls could only be drawn from recent records	Before/during data collection
3	BMI in the clinical model	BMI excluded from the main model	Application of the rule already specified in protocol v2.0 (variables missing in >20% are excluded); observed missingness 53.2%	During data cleaning, before any outcome analysis
4	Five-level ordinal outcome	Not performed	One category (complex hyperplasia without atypia) contained no patients;	During data cleaning

#	Original protocol (v2.0)	Final protocol (v3.0)	Reason	When decided
			chronic endometritis contained three	
5	Bleeding pattern, menopause duration, PCOS, smoking, tamoxifen, HRT, family history, previous biopsy	Removed from the variable list; moved to limitations	Not reliably retrievable from retrospective records	Before/during data collection
6	Dilatation and curettage only	403 D&C plus 5 hysteroscopy-guided procedures; sensitivity analysis SA2 added	Actual composition of the dataset	During data cleaning
7	Nomogram and decision curve analysis	Not performed	Prevalence-dependent measures are not identifiable in an outcome-dependent sample	After primary results were seen
8	PPV/NPV from the sample	Projected at realistic prevalences (Supplementary Table S3)	Same reason as item 7	After primary results were seen
9	NRI/IDI for incremental value	ΔC, DeLong test and calibration slope	NRI/IDI are misleading without calibration and can be positive under the null	After primary results were seen
10	Primary aim: identify independent predictors	Primary aim: test incremental value; ΔC ≥ 0.02 adopted as the threshold for clinical relevance	Direct consequence of the knowledge-gap analysis. The threshold was fixed before the analyses reported in the	**After primary results were seen**

#	Original protocol (v2.0)	Final protocol (v3.0)	Reason	When decided
			manuscript were run, but after the primary results of the original plan had been seen; the manuscript states this and does not describe it as pre-specified	
11	"Enter variables with univariable p < 0.25" plus Models A–E	Model A fixed on clinical rather than statistical grounds; p-based selection abandoned	p-based selection causes overfitting and is not recommended by TRIPOD. The manuscript states that this decision followed the first look at the data and does not describe the model as fixed a priori	After primary results were seen
12	Bonferroni and p < 0.25 together	Bonferroni for the primary tests; Benjamini–Hochberg for exploratory analyses	The two original approaches contradicted each other	After primary results were seen
13	Indices with VIF > 5 tested in separate models	Indices never entered together except in the joint model reported in the article (Model H)	The collinearity is definitional, not statistical (shared lymphocyte denominator); during peer review it was shown to be	During data cleaning

#	Original protocol (v2.0)	Final protocol (v3.0)	Reason	When decided
			exact rather than merely strong (item 27)	
14	Log transformation “will be attempted”	Log transformation mandatory in regression	All indices are heavily right-skewed	During data cleaning
15	A priori power calculation; EPV table; 8–15% expected events	Achieved precision reported instead; EPV table removed	A priori power is not meaningful for a complete-enumeration design; the 8–15% assumption is incompatible with a fixed 1:2 sample	During data cleaning
16	Sensitivity analysis on “insufficient material”	Removed; replaced by the set SA1–SA12	Specimens reported as insufficient (n = 47) were removed from the sampling frame before the draw, so none entered the dataset. SA1–SA8 were specified during data cleaning; SA9–SA11 were added later, after the group asymmetries were identified in internal review; SA12 was added during revision (item 29). All	During data cleaning (SA1–SA8); after primary results were seen (SA9–SA11); during revision (SA12)

#	Original protocol (v2.0)	Final protocol (v3.0)	Reason	When decided
			are described as such in the manuscript	
17	STROBE only	STROBE, TRIPOD and STARD 2015	Required for model evaluation and diagnostic accuracy metrics	After primary results were seen
18	"Simple / complex hyperplasia"	"Hyperplasia without atypia" (WHO 2020)	The original terminology was withdrawn in the 2014 WHO revision	During data cleaning
19	Menopausal subgroup analysis secondary	Stratified analysis central to interpretation	Cases and controls were not matched, so pooled estimates overstate the contrast	After primary results were seen
20	Partial range-check list	Extended range checks plus a differential internal-consistency rule and a record-level repair log	The original list did not cover neutrophils, lymphocytes, monocytes or MPV; sixteen records were consequently detected only by the new rule	During data cleaning
21	Uncited figures for D&C sensitivity, postmenopausal bleeding risk and premalignant/malignant yield	Replaced with verified sourced values	Two of the three original figures were incorrect and none was cited	During data cleaning
22	28 references, two non-existent	All references verified at DOI/PMID level	Reference audit	During data cleaning
23	"Expected sample 200–	Exact figures: 408 (136 cases,	Actual data	Before/during

#	Original protocol (v2.0)	Final protocol (v3.0)	Reason	When decided
	500”	272 controls)		data collection
24	Conflicting counts of 136/272 and 138/276 in the data file	136/272, verified by row count	Internal inconsistency resolved	During data cleaning
25	Performance reported as C-statistic and calibration slope only	Calibration intercept (calibration-in-the-large) and Brier score added, apparent, optimism-corrected and cross-validated	Requested by Reviewer 1. No conclusion is affected; the intercept is reported with the caveat that it reflects the sampling fraction under 1:2 sampling	During peer review, after the submitted results were known
26	Bootstrap optimism correction of the C-statistic and calibration slope	Bootstrap correction (B = 500) extended to the calibration intercept and the Brier score, and 10-fold cross-validation repeated 20 times added, for both the clinical and the joint model	Requested by Reviewer 2. The apparent incremental gain of the index block is shown to be optimism in its entirety	During peer review, after the submitted results were known
27	Joint six-index model tested on six degrees of freedom	Joint model respecified on a maximal identifiable four-index basis (NLR, PLR, MLR, SIRI); degrees of freedom of its likelihood-ratio test corrected from six to four	The six log-indices are exactly linearly dependent, so the design matrix has rank four; identified while answering Reviewer 1. The statistic (χ^2 = 4.48) and the C-statistic are	During peer review, after the submitted results were known

#	Original protocol (v2.0)	Final protocol (v3.0)	Reason	When decided
			unchanged; the p value changes from 0.612 to 0.345 and no conclusion is reversed	
28	Fisher's exact test for all 2 × 2 comparisons	Pearson chi-square computed alongside Fisher's exact test for every categorical comparison, with the minimum expected cell frequency reported (Table S10)	Requested by Reviewer 2. The two tests agree on every variable; Fisher's p values are retained in Table 1 because one comparison has a minimum expected count of 1.67	During peer review, after the submitted results were known
29	Three records that failed the internal-consistency rule retained as entered, without a sensitivity analysis	Sensitivity analysis SA12 added, in which the indices of those three records are also set to missing	An audit of the analysis dataset before resubmission showed that the rule flagged sixteen records rather than the thirteen reported, three having been retained as entered. Every conclusion is unchanged (n = 402, 135 events)	During revision, after the submitted results were known

Table S2. Data-cleaning rule set and record-level repair log

Rule set applied to derive the analysis dataset

1. The raw data file is copied read-only; no operation is performed on it.

2. All continuous variables are converted to numeric type; text entries are reported.
3. Range checks are applied (age 18–100 years; BMI 15–70 kg/m^2; endometrial thickness 1–50 mm; haemoglobin 5–18 g/dL; haematocrit 15–55%; MCV 60–115 fL; RDW 10–30%; leucocytes 1–30, neutrophils 0.5–30, lymphocytes 0.2–10, monocytes 0.05–2.0, eosinophils 0–3, platelets 50–800 ×10^3/μL; MPV 6–15 fL; PDW 8–25%; blood count-to-curettage interval 0–30 days). Every out-of-range cell is flagged.
4. The differential internal-consistency ratio (neutrophils + lymphocytes + monocytes + eosinophils) / leucocytes is computed; records outside 0.85–1.15 are inspected individually.
5. Percentage-entry detection: if applying leucocytes × value / 100 to a flagged cell brings the consistency ratio within 0.85–1.15, the conversion is accepted; otherwise it is rejected.
6. Decimal-shift detection: if dividing or multiplying the value by ten restores the ratio and yields a physiologically plausible result, the correction is accepted.
7. If the responsible cell cannot be identified unambiguously, no repair is attempted and the derived variables are set to missing.
8. If the responsible cell cannot be identified but the discrepancy lies in the leucocyte count, which enters none of the six indices, and every index derived from the record falls inside the cohort distribution, the record is retained as entered; the decision is tested in sensitivity analysis SA12 rather than asserted.
9. All six indices are recalculated from the repaired differential counts; index values as entered in the form are not used.
10. Outlying values with a clinical explanation (high RDW accompanying iron-deficiency anaemia; thick endometrium in postmenopausal carcinoma) are retained and reported as genuine observations, not as outliers.
11. Every repaired record is logged with the action taken and the post-repair verification value.
12. Sensitivity analysis SA3 repeats the primary analysis with all repaired records excluded; sensitivity analysis SA12 additionally sets to missing the indices of the three records that failed the consistency rule but were retained as entered.

Record identifiers. Hospital record numbers have been replaced by sequential labels. R1 to R16 are the sixteen records flagged by the differential internal-consistency rule, numbered in order of appearance; V1 to V4 are the four implausible values in variables outside the index set, which that rule did not flag. The mapping to the source records is retained by the authors and is not reproduced here.

Records flagged by the internal-consistency rule, and their disposition

Record	Leucocytes	Neutrophils / lymphocytes / monocytes / eosinophils as entered	Ratio before	Disposition	Ratio after
R1	10.50	77.20 / 16.50 / 5.40 / 0.60	9.495	Percentages in all four differential fields; converted using the recorded leucocyte count	0.997
R2	14.70	68.50 / 26.30 / 3.80 / 0.17	6.719	Percentages in neutrophils, lymphocytes and monocytes; converted	0.998
R3	11.30	62.40 / 3.46 / 0.49 / 0.29	5.897	Percentage in neutrophils only; converted	0.999
R4	6.16	59.20 / 33.70 / 6.40 / 0.50	16.201	Percentages in all four differential fields; converted	0.998
R5	7.74	68.20 / 24.20 / 0.39 / 0.16	12.009	Percentages in neutrophils and lymphocytes; converted	0.995
R6	6.19	60.90 / 29.80 / 0.42 / 0.11	14.738	Percentages in neutrophils and lymphocytes; converted	0.993
R7	11.14	9.44 / 1.35 / 2.70 / 0.03	1.214	Decimal shift in the monocyte count; divided by ten	0.996
R8	12.20	8.15 / 3.36 / 4.70 / 0.06	1.334	Decimal shift in the monocyte count; divided by ten	0.987
R9	6.94	4.90 / 2.96 / 0.73 / 0.30	1.281	Retained as entered: the excess is over the leucocyte count, which enters no index (NLR 1.66, PLR 101, MLR 0.247, SIRI 1.21, all inside the cohort distribution)	1.281
R10	7.92	5.00 / 30.90 /	4.590	Percentage in	0.997

Record	Leucocytes	Neutrophils / lymphocytes / monocytes / eosinophils as entered	Ratio before	Disposition	Ratio after
		0.42 / 0.03		lymphocytes only; converted	
R11	5.11	3.14 / 0.05 / 0.26 / 0.05	0.685	All six indices set to missing: responsible cell unidentifiable and the derived indices implausible (NLR 62.8, PLR 5520, MLR 5.20)	—
R12	5.11	2.08 / 51.80 / 0.30 / 0.06	10.614	Percentage in lymphocytes only; converted	0.995
R13	0.06	3.44 / 2.12 / 0.36 / 0.11	100.500	Leucocyte count typographically wrong; recomputed as the sum of the differential. Enters no index	1.000
R14	5.99	4.33 / 2.11 / 0.42 / 0.09	1.160	Retained as entered (NLR 2.05, PLR 122, MLR 0.199, SIRI 0.86, all inside the cohort distribution)	1.160
R15	7.58	7.58 / 2.90 / 0.31 / 0.11	1.438	Retained as entered (NLR 2.61, PLR 76, MLR 0.107, SIRI 0.81, all inside the cohort distribution). The recorded leucocyte count equals the recorded neutrophil count, so an error in another field cannot be excluded; SA12 tests this	1.438
R16	8.25	2.47 / 0.82 / 0.82 / 0.01	0.499	All six indices set to missing: responsible cell unidentifiable and the derived index implausible (MLR 1.00 against a cohort range	—

Record	Leucocytes	Neutrophils / lymphocytes / monocytes / eosinophils as entered	Ratio before	Disposition	Ratio after
				of 0.08 to 0.79)	

Values are ×10^3/μL. The consistency ratio is (neutrophils + lymphocytes + monocytes + eosinophils) / leucocytes; the acceptance window was 0.85 to 1.15. Eleven records were repaired, two had all six indices set to missing, and three were retained as entered.

Values changed, by record and variable

Record	Variable	Original	Corrected	Action
R1	Neutrophils	77.2	8.106	percent->absolute
R1	Lymphocytes	16.5	1.732	percent->absolute
R1	Monocytes	5.4	0.567	percent->absolute
R1	Eosinophils	0.6	0.063	percent->absolute
R2	Neutrophils	68.5	10.069	percent->absolute
R2	Lymphocytes	26.3	3.866	percent->absolute
R2	Monocytes	3.8	0.559	percent->absolute
R2	Eosinophils	0.17	0.025	percent->absolute
R3	Neutrophils	62.4	7.051	percent->absolute
R4	Neutrophils	59.2	3.647	percent->absolute
R4	Lymphocytes	33.7	2.076	percent->absolute
R4	Monocytes	6.4	0.394	percent->absolute
R4	Eosinophils	0.5	0.031	percent->absolute
R5	Neutrophils	68.2	5.279	percent->absolute
R5	Lymphocytes	24.2	1.873	percent->absolute
R6	Neutrophils	60.9	3.770	percent->absolute
R6	Lymphocytes	29.8	1.845	percent->absolute
R7	Monocytes	2.7	0.270	decimal shift
R8	Monocytes	4.7	0.470	decimal shift
R10	Lymphocytes	30.9	2.447	percent->absolute
R11	Neutrophils	3.14		unresolvable -> missing
R11	Lymphocytes	0.05		unresolvable -> missing
R11	Monocytes	0.26		unresolvable -> missing
R12	Lymphocytes	51.8	2.647	percent->absolute
R13	Leucocytes	0.06	6.030	typographic

Record	Variable	Original	Corrected	Action
R16	Neutrophils	2.47		unresolvable -> missing
R16	Lymphocytes	0.82		unresolvable -> missing
R16	Monocytes	0.82		unresolvable -> missing
V1	MPV	0.3		implausible -> missing
V2	RDW	3.6		implausible -> missing
V3	MCV	225.0		implausible -> missing
V4	Haematocrit	12.8		implausible -> missing

Records R9, R14 and R15 do not appear in this table because no value in them was changed. V1 to V4 are the four implausible values in variables outside the index set (MPV 0.30 fL, RDW 3.6%, MCV 225 fL, haematocrit 12.8%), each set to missing. Sensitivity analysis SA3 excludes every record touched by repair (n = 17: eleven repaired, two set to missing and the four out-of-index values).

Table S3. Predictive values projected at realistic prevalences

Sensitivity and specificity are those at the Youden cut-off in this sample. Positive and negative predictive values are projected by Bayes' theorem at prevalences of 2% (all women undergoing curettage for abnormal uterine bleeding), 5%, and 11% (postmenopausal bleeding or age ≥ 45 years). They cannot be read off the study sample, in which the prevalence is fixed at 33.3% by design.

Predictor	Cut-off	Sensitivity %	Specificity %	PPV @2 %	NPV @2 %	PPV @5 %	NPV @5 %	PPV @11 %	NPV @11 %
Age	58.0	60.3	87.9	9.2	99.1	20.7	97.7	38.1	94.7
Endometrial thickness	11.0	70.6	71.0	4.7	99.2	11.3	97.9	23.1	95.1
SIRI	0.66	83.1	38.7	2.7	99.1	6.7	97.7	14.3	94.9
NLR	1.91	67.6	49.4	2.7	98.7	6.6	96.7	14.2	92.5
SII	411.62	83.1	33.8	2.5	99.0	6.2	97.4	13.4	94.2
PIV	193.03	74.3	43.9	2.6	98.8	6.5	97.0	14.1	93.2
MLR	0.21	54.4	59.5	2.7	98.5	6.6	96.1	14.2	91.3
PLR	121.43	58.1	50.2	2.3	98.3	5.8	95.8	12.6	90.6

Figure S1. Distribution of the six inflammatory indices by histopathological group

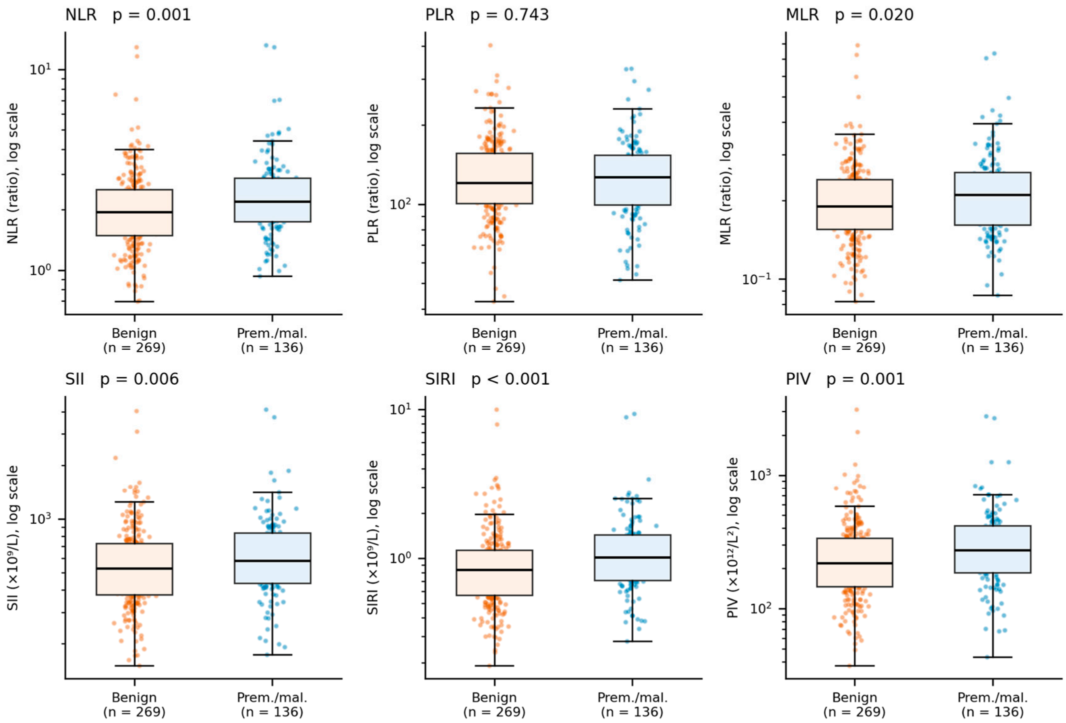

Figure S1. Box plots with individual observations for the six haemogram-derived inflammatory indices in the benign and premalignant/malignant groups (logarithmic y-axis). Boxes show the median and interquartile range; whiskers extend to 1.5 times the interquartile range. p values are from the Mann–Whitney U test. The distributions overlap almost completely, which is the visual counterpart of the areas under the curve of 0.51 to 0.62 reported in Table 3.

Table S4. Pairwise DeLong comparisons among the six indices

Comparison	AUC 1	AUC 2	ΔAUC (95% CI)	p (DeLong)
NLR vs PLR	0.599	0.510	+0.089 (+0.032 to +0.145)	0.002
NLR vs MLR	0.599	0.571	+0.028 (-0.022 to +0.077)	0.272
NLR vs SII	0.599	0.583	+0.015 (-0.019 to +0.049)	0.380
NLR vs SIRI	0.599	0.621	-0.023 (-0.057 to +0.012)	0.201
NLR vs PIV	0.599	0.597	+0.002 (-0.045 to +0.048)	0.944
PLR vs MLR	0.510	0.571	-0.061 (-0.120 to -0.002)	0.042
PLR vs SII	0.510	0.583	-0.073 (-0.121 to -0.026)	0.002
PLR vs SIRI	0.510	0.621	-0.111 (-0.179 to -0.043)	0.001
PLR vs PIV	0.510	0.597	-0.087 (-0.150 to -0.024)	0.007

Comparison	AUC 1	AUC 2	ΔAUC (95% CI)	p (DeLong)
MLR vs SII	0.571	0.583	-0.013 (-0.072 to +0.047)	0.681
MLR vs SIRI	0.571	0.621	-0.050 (-0.092 to -0.008)	0.018
MLR vs PIV	0.571	0.597	-0.026 (-0.080 to +0.028)	0.343
SII vs SIRI	0.583	0.621	-0.038 (-0.076 to +0.001)	0.053
SII vs PIV	0.583	0.597	-0.014 (-0.042 to +0.015)	0.358
SIRI vs PIV	0.621	0.597	+0.024 (-0.001 to +0.050)	0.061

Table S5. Spearman correlation matrix among the six indices

	NLR	PLR	MLR	SII	SIRI	PIV
NLR	1.00	0.56	0.67	0.84	0.84	0.71
PLR	0.56	1.00	0.52	0.70	0.35	0.47
MLR	0.67	0.52	1.00	0.54	0.78	0.64
SII	0.84	0.70	0.54	1.00	0.80	0.89
SIRI	0.84	0.35	0.78	0.80	1.00	0.91
PIV	0.71	0.47	0.64	0.89	0.91	1.00

Table S6. Discrimination stratified by menopausal status

Areas under the curve with DeLong 95% confidence intervals.

Stratum	n (events)	NLR	PLR	MLR	SII	SIRI	PIV	Age	Endometrial thickness
Premenopausal	240 (39 cases)	0.599 (0.497–0.701)	0.461 (0.360–0.561)	0.512 (0.411–0.614)	0.585 (0.488–0.681)	0.603 (0.501–0.705)	0.588 (0.489–0.687)	0.622 (0.519–0.725)	0.772 (0.683–0.861)
Postmenopausal	168 (97 cases)	0.649 (0.562–0.735)	0.588 (0.501–0.676)	0.646 (0.561–0.731)	0.633 (0.546–0.721)	0.664 (0.579–0.748)	0.639 (0.553–0.725)	0.724 (0.645–0.802)	0.789 (0.721–0.858)

Within strata, the clinical model achieved a C-statistic of 0.803 (95% CI 0.714–0.892) among premenopausal women (39 cases in 240) and 0.862 (0.803–0.921) among postmenopausal women (97 cases in 168). Adding ln(SIRI) changed the C-statistic by +0.0035 and +0.0032 respectively; adding ln(NLR) by +0.0113 and

+0.0024. No interaction term between menopausal status and any index approached significance (NLR p = 0.868; PLR p = 0.224; MLR p = 0.143; SII p = 0.995; SIRI p = 0.752; PIV p = 0.731).

Table S7. Sensitivity analyses SA1 to SA10 and SA12

Each analysis repeats the primary comparison of the clinical model with and without the log-transformed index; sample sizes and event counts are given in the row labels. The prefix SA distinguishes these analyses from the supplementary tables, which are numbered S1 to S10. SA1 to SA8 were specified during data cleaning; SA9 to SA11 were added later, after the asymmetries between the groups were identified in internal review; SA12 was added during revision, after an audit of the analysis dataset. SA11 is not a re-run of the primary comparison and is reported separately in Table S7b. SA8 (penalised regression) returned coefficients identical to the unpenalised fit, confirming the absence of separation. For SA12 the p values are likelihood-ratio p values and the ΔC column gives point estimates.

Analysis	Index	aOR (95% CI)	p	ΔC vs clinical model (95% CI)
SA1: cases restricted to carcinoma (n = 362, 93 events)	NLR	0.96 (0.43–2.14)	0.921	+0.0002 (-0.0003 to +0.0006)
SA1: cases restricted to carcinoma (n = 362, 93 events)	PLR	1.10 (0.38–3.20)	0.858	+0.0003 (-0.0004 to +0.0011)
SA1: cases restricted to carcinoma (n = 362, 93 events)	MLR	1.22 (0.45–3.30)	0.695	+0.0000 (-0.0013 to +0.0013)
SA1: cases restricted to carcinoma (n = 362, 93 events)	SII	0.94 (0.47–1.87)	0.855	+0.0002 (-0.0004 to +0.0009)
SA1: cases restricted to carcinoma (n = 362, 93 events)	SIRI	0.99 (0.53–1.85)	0.982	+0.0001 (-0.0001 to +0.0003)
SA1: cases restricted to carcinoma (n = 362, 93 events)	PIV	0.97 (0.57–1.66)	0.925	+0.0002 (-0.0003 to +0.0006)
SA2: hysteroscopy-guided D&C excluded (n = 400, 135 events)	NLR	1.47 (0.77–2.81)	0.246	+0.0027 (-0.0028 to +0.0082)
SA2: hysteroscopy-guided D&C excluded (n = 400, 135 events)	PLR	0.83 (0.37–1.90)	0.664	-0.0000 (-0.0021 to +0.0020)
SA2: hysteroscopy-guided D&C	MLR	1.28	0.547	+0.0011 (-

Analysis	Index	aOR (95% CI)	p	ΔC vs clinical model (95% CI)
excluded (n = 400, 135 events)		(0.58–2.83)		0.0015 to +0.0037)
SA2: hysteroscopy-guided D&C excluded (n = 400, 135 events)	SII	1.16 (0.66–2.04)	0.595	+0.0008 (-0.0017 to +0.0032)
SA2: hysteroscopy-guided D&C excluded (n = 400, 135 events)	SIRI	1.32 (0.80–2.18)	0.270	+0.0019 (-0.0025 to +0.0062)
SA2: hysteroscopy-guided D&C excluded (n = 400, 135 events)	PIV	1.14 (0.74–1.75)	0.554	+0.0008 (-0.0016 to +0.0032)
SA3: all repaired records excluded (n = 390, 126 events)	NLR	1.39 (0.73–2.68)	0.319	+0.0019 (-0.0030 to +0.0067)
SA3: all repaired records excluded (n = 390, 126 events)	PLR	0.85 (0.37–1.95)	0.701	-0.0005 (-0.0025 to +0.0016)
SA3: all repaired records excluded (n = 390, 126 events)	MLR	1.44 (0.64–3.23)	0.371	+0.0011 (-0.0028 to +0.0050)
SA3: all repaired records excluded (n = 390, 126 events)	SII	1.07 (0.60–1.89)	0.822	+0.0001 (-0.0012 to +0.0014)
SA3: all repaired records excluded (n = 390, 126 events)	SIRI	1.29 (0.78–2.13)	0.326	+0.0025 (-0.0020 to +0.0069)
SA3: all repaired records excluded (n = 390, 126 events)	PIV	1.08 (0.70–1.67)	0.718	+0.0001 (-0.0016 to +0.0019)
SA4: adjusted for CBC-to-curettage interval (n = 405, 136 events)	NLR	1.65 (0.86–3.14)	0.131	+0.0042 (-0.0026 to +0.0109)
SA4: adjusted for CBC-to-curettage interval (n = 405, 136 events)	PLR	0.90 (0.40–2.05)	0.810	+0.0003 (-0.0009 to +0.0015)
SA4: adjusted for CBC-to-curettage interval (n = 405, 136 events)	MLR	1.36 (0.62–3.02)	0.445	+0.0005 (-0.0026 to +0.0037)
SA4: adjusted for CBC-to-curettage interval (n = 405, 136 events)	SII	1.23 (0.70–	0.468	+0.0010 (-0.0022 to

Analysis	Index	aOR (95% CI)	p	ΔC vs clinical model (95% CI)
		2.15)		+0.0041)
SA4: adjusted for CBC-to-curettage interval (n = 405, 136 events)	SIRI	1.37 (0.84–2.26)	0.209	+0.0022 (-0.0028 to +0.0071)
SA4: adjusted for CBC-to-curettage interval (n = 405, 136 events)	PIV	1.15 (0.75–1.76)	0.526	+0.0007 (-0.0020 to +0.0034)
SA5: cases restricted to 2025–2026 (n = 323, 54 events)	NLR	1.69 (0.72–3.94)	0.227	+0.0038 (-0.0087 to +0.0162)
SA5: cases restricted to 2025–2026 (n = 323, 54 events)	PLR	0.62 (0.21–1.86)	0.399	+0.0019 (-0.0069 to +0.0107)
SA5: cases restricted to 2025–2026 (n = 323, 54 events)	MLR	2.00 (0.70–5.69)	0.195	+0.0029 (-0.0092 to +0.0150)
SA5: cases restricted to 2025–2026 (n = 323, 54 events)	SII	1.25 (0.60–2.58)	0.548	-0.0001 (-0.0063 to +0.0061)
SA5: cases restricted to 2025–2026 (n = 323, 54 events)	SIRI	1.87 (0.96–3.64)	0.065	+0.0072 (-0.0085 to +0.0229)
SA5: cases restricted to 2025–2026 (n = 323, 54 events)	PIV	1.44 (0.82–2.53)	0.208	+0.0044 (-0.0066 to +0.0155)
SA6: body mass index sub-cohort (n = 191, 72 events)	NLR	3.51 (1.29–9.52)	0.014	+0.0127 (-0.0122 to +0.0376)
SA6: body mass index sub-cohort (n = 191, 72 events)	PLR	0.86 (0.29–2.54)	0.779	-0.0007 (-0.0049 to +0.0035)
SA6: body mass index sub-cohort (n = 191, 72 events)	MLR	3.43 (1.00–11.71)	0.050	+0.0029 (-0.0141 to +0.0199)
SA6: body mass index sub-cohort (n = 191, 72 events)	SII	1.89 (0.84–4.28)	0.126	+0.0037 (-0.0116 to +0.0191)
SA6: body mass index sub-cohort (n = 191, 72 events)	SIRI	3.23 (1.42–7.34)	0.005	+0.0130 (-0.0110 to +0.0369)

Analysis	Index	aOR (95% CI)	p	ΔC vs clinical model (95% CI)
SA6: body mass index sub-cohort (n = 191, 72 events)	PIV	1.91 (1.00–3.66)	0.051	+0.0046 (-0.0123 to +0.0214)
SA7: extreme 1% of each index trimmed (n = 405, 136 events)	NLR	2.01 (0.95–4.25)	0.066	+0.0046 (-0.0040 to +0.0131)
SA7: extreme 1% of each index trimmed (n = 405, 136 events)	PLR	1.03 (0.41–2.60)	0.943	+0.0000 (-0.0006 to +0.0007)
SA7: extreme 1% of each index trimmed (n = 405, 136 events)	MLR	2.07 (0.81–5.27)	0.127	+0.0020 (-0.0035 to +0.0075)
SA7: extreme 1% of each index trimmed (n = 405, 136 events)	SII	1.41 (0.73–2.71)	0.306	+0.0013 (-0.0034 to +0.0061)
SA7: extreme 1% of each index trimmed (n = 405, 136 events)	SIRI	1.64 (0.92–2.93)	0.093	+0.0028 (-0.0035 to +0.0091)
SA7: extreme 1% of each index trimmed (n = 405, 136 events)	PIV	1.22 (0.74–2.01)	0.431	+0.0001 (-0.0029 to +0.0030)
SA8: penalised (Firth-type) regression (n = 405, 136 events)	NLR	1.61	—	penalised fit
SA8: penalised (Firth-type) regression (n = 405, 136 events)	PLR	0.90	—	penalised fit
SA8: penalised (Firth-type) regression (n = 405, 136 events)	MLR	1.35	—	penalised fit
SA8: penalised (Firth-type) regression (n = 405, 136 events)	SII	1.23	—	penalised fit
SA8: penalised (Firth-type) regression (n = 405, 136 events)	SIRI	1.37	—	penalised fit
SA8: penalised (Firth-type) regression (n = 405, 136 events)	PIV	1.16	—	penalised fit
SA9: pandemic-period cases (2021–2022) excluded (n = 372, 103 events)	NLR	1.34 (0.67–2.66)	0.410	+0.0005 (-0.0046 to +0.0055)
SA9: pandemic-period cases (2021–2022) excluded (n = 372, 103 events)	PLR	0.83 (0.34–2.04)	0.683	+0.0003 (-0.0017 to +0.0024)

Analysis	Index	aOR (95% CI)	p	ΔC vs clinical model (95% CI)
SA9: pandemic-period cases (2021–2022) excluded (n = 372, 103 events)	MLR	1.47 (0.62–3.50)	0.378	+0.0007 (-0.0041 to +0.0055)
SA9: pandemic-period cases (2021–2022) excluded (n = 372, 103 events)	SII	1.07 (0.59–1.95)	0.814	+0.0002 (-0.0015 to +0.0020)
SA9: pandemic-period cases (2021–2022) excluded (n = 372, 103 events)	SIRI	1.31 (0.77–2.24)	0.318	+0.0010 (-0.0044 to +0.0063)
SA9: pandemic-period cases (2021–2022) excluded (n = 372, 103 events)	PIV	1.12 (0.71–1.78)	0.622	+0.0002 (-0.0026 to +0.0030)
SA10: restricted to the common age range, 45–65 years (n = 233, 81 events)	NLR	1.36 (0.63–2.93)	0.436	+0.0030 (-0.0044 to +0.0104)
SA10: restricted to the common age range, 45–65 years (n = 233, 81 events)	PLR	0.82 (0.31–2.22)	0.702	+0.0010 (-0.0028 to +0.0047)
SA10: restricted to the common age range, 45–65 years (n = 233, 81 events)	MLR	1.48 (0.58–3.75)	0.412	+0.0024 (-0.0039 to +0.0087)
SA10: restricted to the common age range, 45–65 years (n = 233, 81 events)	SII	0.98 (0.50–1.91)	0.950	+0.0003 (-0.0007 to +0.0013)
SA10: restricted to the common age range, 45–65 years (n = 233, 81 events)	SIRI	1.22 (0.68–2.19)	0.496	+0.0021 (-0.0040 to +0.0082)
SA10: restricted to the common age range, 45–65 years (n = 233, 81 events)	PIV	1.01 (0.61–1.67)	0.970	-0.0002 (-0.0010 to +0.0007)
SA12: the three records retained as entered set to missing (n = 402, 135 events)	NLR	1.64 (0.86–3.11)	0.130	+0.0045
SA12: the three records retained as entered set to missing (n = 402, 135 events)	PLR	0.90 (0.40–2.04)	0.797	+0.0000
SA12: the three records retained as entered set to missing (n = 402, 135 events)	MLR	1.28 (0.58–2.82)	0.540	+0.0012
SA12: the three records retained as	SII	1.23	0.464	+0.0017

Analysis	Index	aOR (95% CI)	p	ΔC vs clinical model (95% CI)
entered set to missing (n = 402, 135 events)		(0.71–2.14)		
SA12: the three records retained as entered set to missing (n = 402, 135 events)	SIRI	1.35 (0.82–2.21)	0.236	+0.0022
SA12: the three records retained as entered set to missing (n = 402, 135 events)	PIV	1.13 (0.74–1.73)	0.564	+0.0009

Table S7b. Sensitivity analysis SA11: characteristics of women with and without a recorded body mass index

Denominators. The comparisons of age, endometrial thickness, menopausal status, comorbidity and case status use all 408 women (191 with a recorded body mass index, 217 without). The NLR and SIRI rows use the 405 women with complete index data, so the column totals for those two rows are marginally smaller. Women with a recorded BMI had a higher NLR and a thicker endometrium than those without, identifying the sub-cohort as selected rather than representative; age, menopausal status and comorbidity did not differ. Within this sub-cohort, removing BMI from the model barely changed the index estimates (NLR adjusted OR 3.32, 1.25–8.79, p = 0.016 without BMI versus 3.51, 1.29–9.52, p = 0.014 with BMI; SIRI 2.90, 1.32–6.39, p = 0.008 versus 3.23, 1.42–7.34, p = 0.005), indicating that the signal is a property of the sub-cohort rather than of adjustment for obesity.

Variable	BMI recorded (n = 191)	BMI not recorded (n = 217)	p
Age (years)	48.00 (42.00–59.00)	49.00 (44.00–59.00)	0.212
Endometrial thickness (mm)	10.00 (7.00–15.00)	9.00 (6.00–14.00)	0.037
SIRI	0.88 (0.62–1.27)	0.87 (0.61–1.21)	0.478
NLR	2.08 (1.70–2.84)	1.99 (1.48–2.48)	0.010
Postmenopausal, n (%)	81 (42.4)	87 (40.1)	0.687
Diabetes, n (%)	33 (17.3)	29 (13.4)	0.333
Hypertension, n (%)	43 (22.5)	42 (19.4)	0.465
Case, n (%)	72 (37.7)	64 (29.5)	0.092

Table S8. Missing data by source

Values missing in the source records are distinguished from values set to missing by the data-cleaning rules; only the latter appear in the repair log of Table S2.

Variable	Missing in source records	Set to missing during cleaning	Total missing	n analysed	% missing
Age	0	0	0	408	0.0
Body mass index	217	0	217	191	53.2
Endometrial thickness	0	0	0	408	0.0
Haemoglobin	5	0	5	403	1.2
Haematocrit	0	1	1	407	0.2
MCV	0	1	1	407	0.2
RDW	0	1	1	407	0.2
Leucocytes	0	0	0	408	0.0
Neutrophils	0	2	2	406	0.5
Lymphocytes	1	2	3	405	0.7
Monocytes	0	2	2	406	0.5
Platelets	0	0	0	408	0.0
MPV	0	1	1	407	0.2
PDW	1	0	1	407	0.2
NLR	0	3	3	405	0.7
PLR	0	3	3	405	0.7
MLR	0	3	3	405	0.7
SII	0	3	3	405	0.7
SIRI	0	3	3	405	0.7
PIV	0	3	3	405	0.7

Table S9. Temporal drift probe within the case group (2021–2026)

Spearman correlation between calendar year of curettage and each index or cell count, restricted to the 136 cases. p values are given uncorrected and after Benjamini–Hochberg adjustment across the eleven tests.

Variable	n	Spearman rho (95% CI)	p (uncorrected)	p (BH-adjusted)
NLR	136	+0.010 (-0.159 to +0.178)	0.911	0.911
PLR	136	-0.056 (-0.222 to +0.114)	0.519	0.714
MLR	136	+0.061 (-0.109 to +0.227)	0.482	0.714
SII	136	+0.021 (-0.148 to +0.189)	0.806	0.887
SIRI	136	+0.100 (-0.070 to +0.264)	0.247	0.664
PIV	136	+0.085 (-0.085 to +0.249)	0.327	0.664

Variable	n	Spearman rho (95% CI)	p (uncorrected)	p (BH-adjusted)
Neutrophils	136	+0.098 (-0.072 to +0.262)	0.259	0.664
Lymphocytes	136	+0.079 (-0.091 to +0.244)	0.362	0.664
Monocytes	136	+0.205 (+0.038 to +0.361)	0.017	0.187
Platelets	136	+0.023 (-0.146 to +0.190)	0.793	0.887
Leucocytes	136	+0.154 (-0.015 to +0.314)	0.074	0.407

Table S10. Chi-square and Fisher's exact test results for all categorical variables

Chi-square values are Pearson's statistic without continuity correction, on one degree of freedom. The minimum expected cell frequency is the smallest of the four expected counts of the 2 × 2 table. Fisher's exact p values are those reported in Table 1 of the main text. The two tests agree on every variable. For hysteroscopy-guided dilatation and curettage the minimum expected frequency is 1.67, so the chi-square approximation is not appropriate for that comparison and the exact test is required; Fisher's exact test is valid for all five variables and is therefore the test reported throughout Table 1.

**Variable**	**Benign (n = 272)**	**Premalignant/malignant (n = 136)**	**Minimum expected count**	**χ^2 (1 df)**	**p (chi-square)**	**p (Fisher's exact)**
Postmenopausal status	71 (26.1)	97 (71.3)	56.00	76.55	<0.001	<0.001
Diabetes mellitus	17 (6.2)	45 (33.1)	20.67	50.68	<0.001	<0.001
Hypertension	22 (8.1)	63 (46.3)	28.33	80.37	<0.001	<0.001
Nulliparity	11 (4.0)	10 (7.4)	7.00	2.03	0.154	0.161
Hysteroscopy-guided D&C	4 (1.5)	1 (0.7)	1.67	0.40	0.525	0.669

Values in the group columns are n (%). Analyses used Python 3.11 (SciPy): scipy.stats.chi2_contingency with correction=False for the chi-square test and the expected counts, and scipy.stats.fisher_exact for the exact test.
